# Intergenomic Comparisons Highlight Modularity of the Denitrification Pathway and Underpin the Importance of Community Structure for N_2_O Emissions

**DOI:** 10.1371/journal.pone.0114118

**Published:** 2014-12-01

**Authors:** Daniel R. H. Graf, Christopher M. Jones, Sara Hallin

**Affiliations:** Department of Microbiology, Swedish University of Agricultural Sciences, Uppsala, Sweden; University of Florida, United States of America

## Abstract

Nitrous oxide (N_2_O) is a potent greenhouse gas and the predominant ozone depleting substance. The only enzyme known to reduce N_2_O is the nitrous oxide reductase, encoded by the *nosZ* gene, which is present among bacteria and archaea capable of either complete denitrification or only N_2_O reduction to di-nitrogen gas. To determine whether the occurrence of *nosZ*, being a proxy for the trait N_2_O reduction, differed among taxonomic groups, preferred habitats or organisms having either NirK or NirS nitrite reductases encoded by the *nirK* and *nirS* genes, respectively, 652 microbial genomes across 18 phyla were compared. Furthermore, the association of different co-occurrence patterns with enzymes reducing nitric oxide to N_2_O encoded by *nor* genes was examined. We observed that co-occurrence patterns of denitrification genes were not randomly distributed across taxa, as specific patterns were found to be more dominant or absent than expected within different taxonomic groups. The *nosZ* gene had a significantly higher frequency of co-occurrence with *nirS* than with *nirK and* the presence or absence of a *nor* gene largely explained this pattern, as *nirS* almost always co-occurred with *nor*. This suggests that *nirS* type denitrifiers are more likely to be capable of complete denitrification and thus contribute less to N_2_O emissions than *nirK* type denitrifiers under favorable environmental conditions_._ Comparative phylogenetic analysis indicated a greater degree of shared evolutionary history between *nosZ* and *nirS.* However 30% of the organisms with *nosZ* did not possess either *nir* gene, with several of these also lacking *nor*, suggesting a potentially important role in N_2_O reduction. Co-occurrence patterns were also non-randomly distributed amongst preferred habitat categories, with several habitats showing significant differences in the frequencies of *nirS* and *nirK* type denitrifiers. These results demonstrate that the denitrification pathway is highly modular, thus underpinning the importance of community structure for N_2_O emissions.

## Introduction

Managing nitrogen (N) is one of the major environmental challenges for the 21^st^ century [1], [2]. Of special concern is the mitigation of nitrous oxide (N_2_O) due to its status as a potent greenhouse gas and the predominant ozone depleting compound [3], [4]. While there are multiple sources of atmospheric N_2_O, the majority is produced by microbial transformations of reactive N in fertilized agricultural soil [5]–[7].

The only known sink for N_2_O in Earth's troposphere is microbial reduction to di-nitrogen (N_2_), catalyzed by the N_2_O reductase encoded by the *nosZ* gene. This unique enzyme is found among a wide range of bacterial and archaeal taxa capable of complete denitrification, an anaerobic respiratory process in which soluble nitrate (NO_3_^−^) or nitrite (NO_2_^−^) is reduced to the gases nitric oxide (NO), N_2_O and N_2_ via a series of enzymatic steps. It has been suggested that denitrification is a modular pathway, in that an organism may not always possess the full set of enzymes and thus perform only a subset of steps within the pathway [8]. Bacteria carrying *nosZ* genes that reduce N_2_O to N_2_ yet lack the other denitrification genes have been described [9]–[12], and have recently been shown to be potentially important consumers of N_2_O produced by denitrification or other N-cycling processes [13]. On the other hand, denitrifiers can also lack the *nosZ* gene [14], and thus produce N_2_O as a terminal product. It was recently shown that manipulation of the proportion of denitrifiers with the *nosZ* gene in soil microcosms significantly affected the ratio of N_2_O:N_2_ production, establishing a causal link between denitrifier community composition and potential N_2_O emissions [15]. Thus, the regulation of N_2_O emissions ultimately has a genetic basis.

Whether the genetic potential to produce or reduce N_2_O is more conserved among specific microbial taxa or functional groups of denitrifiers implies that the composition of the microbial community is not trivial from an ecosystem perspective, however this is not known. Denitrifiers can be divided into two functional groups based on whether they possess the copper binding dissimilatory nitrite reductase (NirK) or the cytochrome *cd*_1_ variant (NirS) that catalyze the reduction of NO_2_^−^ to NO; the key reaction that defines denitrification [8], [16]. While the two enzymes perform the same function in the denitrification pathway, they are non-homologous and were thought to be mutually exclusive in the genomes of denitrifying organisms [14]. Several studies have shown that communities of denitrifiers with NirK respond differently to environmental gradients than those with NirS [17]–[21], which supports the hypothesis that the two communities occupy different ecological niches [22]. Thus, *nosZ* co-occurrence patterns in relation to denitrifying organisms with one or the other type of nitrite reductase can provide additional insight on the importance of denitrifier community composition for N_2_O reduction in different ecosystems.

To understand the ecological implications of whether NO_2_^−^ reduction is performed by NirK or NirS denitrifiers, the link to the enzymatic step reducing NO to N_2_O needs to be considered. This reaction may be performed by a number of different enzymes that vary in structure and evolutionary relationships, including periplasmic associated flavodiiron, flavorubredoxin, and cytochrome-c type proteins, as well as several variants of the membrane-bound heme-copper oxidases [23]. Whereas the periplasmic enzymes are generally associated with response to nitrosative stress [23], [24], respiratory NO reduction to N_2_O in the denitrification pathway is performed by two variants of the heme-copper oxidase type NO reductases in bacterial and archaeal denitrifiers, encoded by the *cnorB* or *qnorB* genes [25]. Homologs of the *qnorB* gene have also been observed in non-denitrifying pathogenic species as well as anaerobic denitrifying methanotrophs, and are believed to be involved in detoxification [26] and dismutation of NO to N_2_ and O_2_ [27], respectively. In fungal denitrifiers, NO reduction is performed by the P450-type nitric oxide reductase (P450_nor_), however this enzyme is not considered to be involved in energy conservation [28]. Regardless of the cellular role of each enzyme, total N_2_O production from a given ecosystem could be considered a sum of the activities of each N_2_O-genic NO reductase, although the contribution of *qnorB* and *cnorB* likely outweighs that of other Nor-types due to their prominent role in anaerobic respiration [24].

The aim of our study was to determine whether patterns of co-occurrence of *nosZ*, being a proxy for the trait N_2_O reduction, differed among *nirK* and *nirS* type denitrifiers, how this relates to taxonomic affiliation, and whether these patterns could be explained by the presence of a *nor* gene encoding one of the canonical NO reductases or the p450_nor_ variant for fungal denitrifiers. This was investigated by examining the distribution of the genes *nirK*, *nirS, nor* and *nosZ* in 652 publicly available microbial genomes across 18 phyla. The increasing number of genomes from organisms found in a diverse range of environments [29] also allowed for an assessment of the modularity of the denitrification pathway in relation to preferred habitat. Furthermore, we examined how conserved the trait of N_2_O reduction is from an evolutionary perspective.

## Materials and Methods

### Data acquisition

A local database was constructed by downloading all 4135 draft and completed microbial genome nucleotide sequences available (November 2012) at the National Center for Biology Information (NCBI, www.ncbi.nlm.nih.gov). To ensure that homology searches were as comprehensive as possible, a two-step procedure was performed for each gene. First, an initial TBLASTN search [30] of the online NCBI microbial genomes database (www.ncbi.nlm.nih.gov/sutils/genom_table.cgi) was performed using translated *nirS*, *nirK*, and *nosZ* gene sequences from either *Paracoccus denitrificans* PD1222 or *Bradyrhizobium japonicum* USDA110 as queries. Resulting hits were then translated to amino acid sequences and aligned using SATÉ v2.2.3 [31] with MAFFT [32] as aligner, MUSCLE as merger and RAxML [33] as the tree estimator. Gene identity of the retrieved sequences was confirmed by examining the amino acid alignments in relation to characterized homologs, with emphasis on conserved positions crucial for protein functioning and phylogenetic inference (see below). The resulting amino acid alignments of *nirK, nirS* and *nosZ*, with 477, 150 and 282 sequences, respectively, were then used to create Position Specific Score Matrices (PSSM) [34] for conducting a more comprehensive PSI-TBLASTN search of the downloaded database. Truncated sequences and sequences with stop codons were excluded, and redundancy in the data set was reduced by eliminating different strains of the same species with identical *nirK, nirS* and *nosZ* amino acid sequences. Strains with identical sequences were kept when a unique co-occurrence pattern of denitrification genes was observed, or when the sequence of another denitrification gene was not identical, resulting in a dataset of 652 organisms (see Table S1 for species name, NCBI taxon ID, project name). We then searched the final set of genomes for homologues of the *qnorB* and *cnorB* variants of the NO-reductase. This was performed in a similar manner as described for the *nir* and *nos* genes, with the exception that the PSSM was generated by downloading the 10 most diverse representative cNorB and qNorB amino acid sequences from the NCBI conserved domains database (http://www.ncbi.nlm.nih.gov/Structure/cdd/cdd.shtml) to allow for an equal representation of both variants within the initial PSSM. For the eukaryotic species, the amino acid sequence for the P450_nor_ from *Fusarium oxysporum* [35] was used as a query for TBLASTN searches of each fungal genome, and the resulting hits were aligned to the query sequence to both correctly identify P450_nor_ based on previously described conserved amino acid positions [28], as well as to aid in assembly of exons.

Small subunit (SSU) rRNA gene sequences corresponding to the organisms were retrieved from the local genome database using Infernal [36]. In cases where there was more than one SSU rRNA gene sequence in a genome, the longest sequence was chosen. Taxonomic assignment was based on NCBI classification, which was verified by classification of SSU sequences using the SILVA database [37]. In addition, habitat and isolation source was either downloaded from the Genomes online database (GOLD, 2012 November 15, www.genomesonline.org/) [29] or searched for in NCBI using the taxon ID of the respective genome and looking at connected publications when available.

### Phylogenetic tree reconstruction

Preliminary amino acid alignments of full-length gene sequences of *nirK, nirS, norB, nosZ*, and P450_nor_ were created using the MUSCLE alignment algorithm [38] in the Geneious bioinformatics software suite (version 5.6.1, Biomatters, Auckland, New Zealand) with default settings. With these alignments as input, the most suitable substitution models were inferred by ProtTest v3.2 [39], those being LG+Γ for *nirK* and *nosZ*, LG+Γ+F for *norB* and *nirS* [40], and WAG+Γ+F for P450_nor_. Subsequently, new alignments were inferred using SATÉ, and after manual inspection of the resulting alignments maximum likelihood (ML) phylogenetic trees were inferred using 30 RAxML tree searches and selecting the best likelihood topology, followed by bootstrap analysis with 250 replicates. Small subunit rRNA sequences were aligned using SINA [41]. After manual improvement of the resulting alignment, the phylogeny was determined using 50 tree searches in RAxML with GTRCAT as the nucleotide substitution model followed by 500 bootstrap replicates. All trees were displayed and annotated using the Interactive Tree of Life (iTOL) online tool [42].

### Phylogenetic distribution and conservation of nosZ among nirK- and nirS denitrifiers

To compare the phylogenetic pattern of N_2_O reduction between denitrifiers with *nirK* and *nirS*, the *nosZ* gene was used as a proxy for the capacity of an organism to reduce N_2_O and mapped onto the corresponding taxa within the *nirK* and *nirS* phylogenies. Quantification of the phylogenetic patterns of *nosZ* distribution for both genes was performed using Fritz and Purvis' [43] measure of phylogenetic signal strength (*D*), implemented in the ‘caper’ package within the R statistical programming environment (R foundation for statistical computing, Vienna, Austria). This statistic measures whether a trait is highly clumped (*D*<0), displays clumping patterns due to Brownian evolutionary processes (*D*∼0) or random distribution patterns (*D* = 1), or is highly over-dispersed (*D*>1) across a given phylogeny. In order to also determine the degree to which the ability to reduce N_2_O is conserved amongst *nirK* and *nirS* denitrifiers, we used the phylogenetic metric ConsenTRAIT [44] to calculate trait depths (τ_D_) of *nosZ* as a trait on *nirK* and *nirS* gene trees in the R environment. Trait depth is a measure of the average distance between organisms exhibiting the trait and their last common ancestor, such that increasing values of τ_D_ indicate more conserved traits.

### Gene co-occurrence patterns in relation to taxa and habitat

The genomic co-occurrence patterns of *nirK*, *nirS, nor* and *nosZ* were investigated in relation to taxonomic affiliation based on the 16S rRNA gene phylogeny at different taxonomic ranks. In order to determine whether the co-occurrence patterns of *nir* and *nos* genes were randomly distributed among taxa, contingency tables of co-occurrence patterns by taxonomic affiliation at the phylum, class, order and family was performed, followed by Chi-squared goodness of fit tests in the R environment. Lower taxonomic ranks were not examined due to low frequencies (<5) in the majority of cells. In addition, the standardized residuals of the resulting cell values were examined to distinguish taxa that differed significantly from the expected values for each pattern of co-occurrence, and presented as a mosaic plot generated in the R environment using the ‘vcd’ package. For each possible combination of *nosZ* and *nir* genes, the fraction harboring a *nor* gene was determined.

The co-occurrence patterns of *nirK*, *nirS* and *nosZ* were also correlated with available information on the organism's lifestyle as determined by habitat preference and for each possible combination, the fraction harboring a *nor* gene was determined. Information on habitat preference could be retrieved for 626 organisms that were grouped into eight general habitat categories: wastewater treatment plants, extremophilic, marine systems, fresh water systems, soil, plant, animal, and food associated organisms, and those found in multiple habitats. Here, extremophilic represents habitats for thermo-, psychro-, halo-, acido- and alkalophiles as well as oil degrading, radiation resistant and arsenic resistant organisms. The plant and animal associated organisms are specified as being directly associated to a plant or animal host, whereas food associated are those used in food production. Organisms isolated from multiple sources without any specific preferences were lumped into the ‘multiple habitats’ category. The different categories of lifestyle versus the gene occurrence patterns were tested in the same way as for taxa affiliation.

## Results

### Genomic nirK, nirS, nor and nosZ co-occurrences

**Figure 1:**
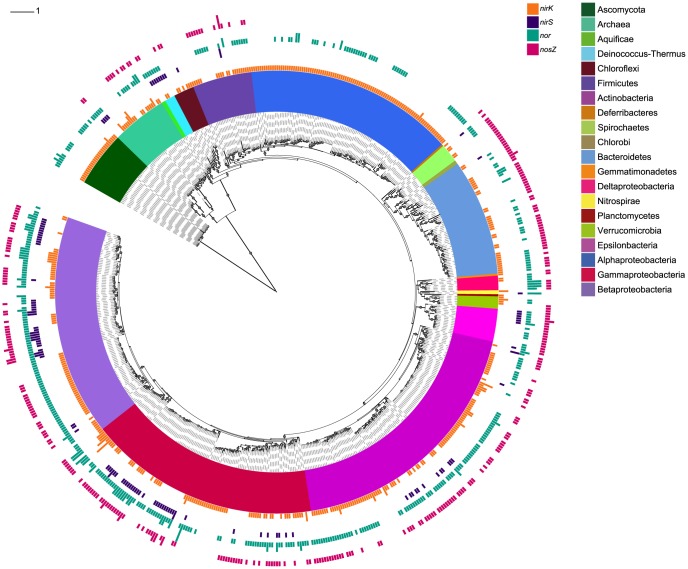
Maximum likelihood phylogeny of full-length 16S/18S rRNA sequences from 652 organisms with denitrification genes. The inner colored ring represents taxonomic affiliation as indicated by the legend. The four outer bar-chart rings show the presence of *nirK* (orange), *nirS* (purple), *nor* (turquoise) and *nosZ* (magenta). Bar height represents the number of copies (≤4). Bootstrap values >70% are indicated by grey circles, and the scale bar denotes nucleotide substitution rate (GTR+Γ). Classification is based on the SILVA database with denomination according to NCBI taxonomy. For NCBI taxon ID number and project name, see Table S1.

**Figure 2:**
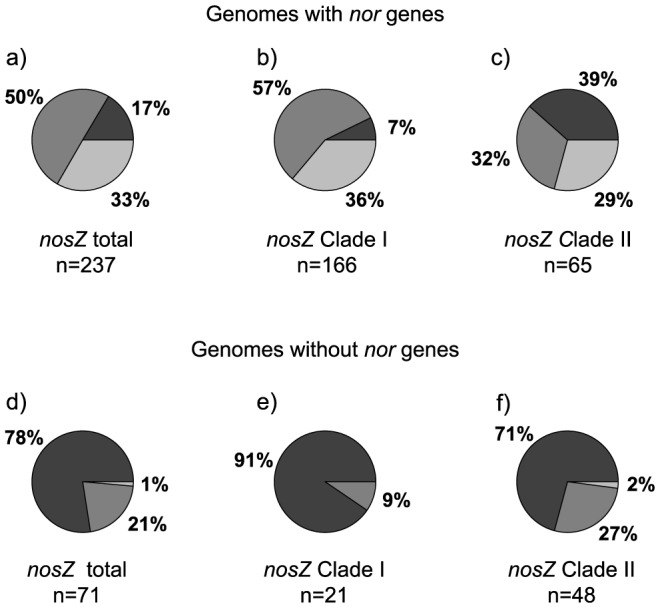
Co-occurrence of *nosZ* with *nirK* or *nirS* in genomes with and without *nor* genes. Percentage of genomes with only *nosZ* (black), *nosZ* and *nirK* (dark grey) and *nosZ* and *nirS* (light grey) among organisms a) harboring *nosZ* and *nor*, b) within *nosZ* Clade I with *nor*, c) within *nosZ* Clade II with *nor*, d) harboring *nosZ* without *nor*, e) within *nosZ* Clade I without *nor*, and f) within *nosZ* Clade II without *nor*. Six genomes that have both *nirS* and *nirK* in addition to *nosZ* are excluded as well as eight halophilic Archaea that group outside Clade I and II in b) and c).

In total, 652 organisms from all three domains of life were found to harbor denitrification genes (Fig. 1; for greater detail see Fig. S1 and Table S1). Of these, 458 and 110 possessed a *nirK* or *nirS* homolog, respectively, including ten genomes that harbored both *nir* genes. As stated earlier, we limited our search of NO-reductases to the *cnorB* and *qnorB* variants encoding NorB found in the canonical denitrification pathway, as well as the p450_nor_ variant for fungal denitrifiers. This resulted in a total of 431 organisms with a *nor* gene, whereas 314 genomes were found to possess a *nosZ* gene. Interestingly, a large part of the *nirK* organisms (35%) had a truncated pathway lacking a canonical *nor* and a majority (70%) did not have *nosZ*. By contrast, only 3.6% of the *nirS*-type denitrifiers lacked *nor* and 20% did not have a *nos*Z gene. Of all organisms harboring *nosZ*, 30% did not possess any *nir* genes and about 24% did not have *nor*. In general, genomes containing both *nosZ* and *nor* also had a *nir* gene, whereas organisms with *nosZ* that did not have a *nor* gene also lacked a *nir* gene (Fig. 2a and d).

It was recently established that the N_2_O reductase can be divided into two phylogenetic clades (I and II; Fig. S5), with clade II being a previously unaccounted, yet environmentally abundant lineage [45], [46]. Of the genomes with *nosZ*, 187 and 113 harbored lineages from clade I and II, respectively and 89 and 56% of these also had a *nor* gene. Among those with *nor*, organisms that also possessed *nirK* dominated in clade I, whereas in clade II the co-occurrence patterns were evenly distributed (Fig. 2b and c, respectively). By contrast, *nosZ*-harboring genomes lacking *nor* most often also lacked *nir* genes (Fig. 2d–f).

### Analysis of denitrification gene phylogeny and nosZ conservation

Phylogenetic analysis of amino acid alignments resulted in well-supported tree topologies, with 48%, 63%, 62% and 66% of nodes having bootstrap support greater than 70% for *nirK*, *nirS*, *norB*, and *nosZ* phylogenies, respectively (Figs. S2–S5). Similar to previous reports, the *nirK* phylogeny (Fig. S2) could be divided into two major clusters [47], each consisting of sequences from a highly diverse range of phyla with no clear correspondence with organismal phylogeny [14]. While the occurrence of organisms with *nosZ* was dispersed throughout the overall *nirK* phylogeny, the Group 1 cluster had a noticeably higher number of organisms with *nosZ* than the Group 2 cluster, which was dominated by sequences from genomes that lack the *nosZ* gene. Similarly, *nirK* from the eukaryotic genomes formed a well-supported monophyletic clade that also corresponded with the lack of *nosZ* genes. This pattern of a more clumped occurrence of *nosZ* across the *nirK* phylogeny was confirmed by the calculated phylogenetic signal strength (*D*) of 0.335, which indicates a moderately clumped distribution that was significantly different from both random (*p*<0.001) and Brownian distributions (*p* = 0.002) of phylogenetic trait patterns.

The *norB* phylogeny could be clearly divided into *cnorB* and *qnorB* variants, with a diverse range of bacterial and archaeal phyla found within each clade (Fig. S4). However, a greater proportion of organisms with *nirS* were found within the *cnorB* clade, whereas those with *nirK* and *nosZ* were dispersed more equally across both clades.

In contrast to *nirK* and *norB*, the *nirS* and *nosZ* phylogenies (Figs. S2 and S3) were more congruent to taxonomic affiliation on the phylum level, with some exceptions observed within each tree. While the few *nirS* genomes lacking *nosZ* appeared to be randomly distributed throughout the *nirS* phylogeny, sequences from genomes lacking *nosZ* within the phyla Deinococcus-Thermus and Chloroflexi each formed monophyletic clades within the *nirS* phylogeny. This was reflected in the resulting phylogenetic signal strength (*D* = 0.431) that was also significantly non-random (*p*<0.001) and did not correspond to a Brownian process (*p* = 0.02), yet was slightly less clumped than the pattern of *nosZ* distribution observed for the *nirK* phylogeny. Additional analysis using the ConsenTRAIT metric (τ_D_), performed using *nosZ* occurrence as a proxy for the trait N_2_O reduction on both the *nirS* and *nirK* phylogenies, indicated a higher degree of evolutionary conservation of *nosZ* with *nirS* (τ_D_ = 0.089) than *nirK* (τ_D_ = 0.043).

### Gene co-occurrence patterns in relation to taxa

The SSU tree showing the distribution of *nir, nor* and *nos* genes (Fig. 1) indicated that patterns of gene co-occurrence were not randomly distributed across different taxonomic groups. Chi-squared tests of taxonomy-based contingency tables confirmed that the patterns *nirK* only (K-type), *nirK+nosZ* (KZ-type), *nirK*+*nirS* (KS-type), *nirK*+*nirS*+*nosZ* (KSZ-type), *nirS* only (S-type), *nirS+nosZ* (SZ-type) and only *nosZ* (Z-type) were indeed not randomly distributed amongst groupings at either the phylum, class, or order levels (*P*<0.001; Tables 1, S2 and S3). Calculation of standardized χ^2^ residuals highlighted several taxonomic groups that significantly contributed to the resulting chi-squared values. Within the largest represented phylum, the proteobacteria, nearly half of all organisms had a complete denitrification pathway including *nir*, *nor* and *nosZ* and the majority had *nir* and *nor* (Table 1). The K and KZ patterns dominated among the Alphaproteobacteria, largely due to the overrepresentation of these patterns within the order Rhizobiales (Table S3). However, the negative χ^2^ residual values observed for the abundance of K-only patterns indicates that proteobacteria in general are less likely to have a partial pathway, particularly Beta- and Gammaproteobacteria (Tables 1 and S2). This is further supported by the abundances of SZ and KZ patterns, which were higher than expected by chance within the proteobacteria, as well as the high percentage of organisms harboring *nor* genes for each pattern. The SZ pattern was twice as frequent as the KZ or Z patterns among the Gammaproteobacteria, which could be attributed to the Pseudomonadales. Within the Betaproteobacteria, the SZ- and KZ-types were equally represented, while Z-types were significantly underrepresented. SZ-type organisms within the Betaproteobacteria were mainly represented by the orders Burkholderiales and Rhodocyclales, whereas the KZ-type Betaproteobacteria were in turn primarily associated with the orders Neisseriales and Burkholderiales. The K-and Z-type Alpha- and Gammaproteobacteria lacked *norB* to a significantly greater extent than those within the Beta- and Deltaproteobacteria.The Ascomycota and Actinobacteria phyla were exclusively K-type organisms, whereas Z-type and KZ-type organisms were both overrepresented within the Bacteroidetes phylum (Table 1). A third of all KZ-type Bacteroidetes did not have a *norB* gene, which can be attributed to the Flavobacteriales (Tables 1 and S3).

**Table 1:** Frequency table of *nirK* (K), *nirS* (S) and *nosZ* (Z) co-occurrence types and taxonomic affiliation at phylum level as well as the percentage of organisms within each phylum that also harbor a *nor* gene.

Phylum	K	%*nor*	KS	%*nor*	KSZ	%*nor*	KZ	%*nor*	S	%*nor*	SZ	%*nor*	Z	%*nor*
Actinobacteria	102↑	42	0	-	0	-	0↓	-	0	-	0↓	-	0↓	-
Aquificae	0	-	0	-	0	-	0	-	0	-	2↑	100	0	-
Ascomycota	27↑	67	0	-	0	-	0↓	-	0	-	0	-	0↓	-
Bacteroidetes	8↓	100	0	-	1	0	24↑	67	0	-	0↓	-	24↑	17
Chlorobi	0	-	0	-	0	-	0	-	0	-	0	-	2↑	0
Chloroflexi	5	0	0	-	0	-	1	0	2↑	0	0	-	2	0
Crenarchaeota	0	-	0	-	0	-	0	-	2↑	100	2↑	100	0	-
Deferribacteres	0	-	0	-	0	-	0	-	0	-	0	-	1↑	0
Deinococcus-Thermus	0↓	-	2↑	100	0	-	0	-	3↑	100	0	-	0	-
Euryarchaeota	5	60	0	-	0	-	4	100	0	-	0	-	5↑	0
Firmicutes	16	25	0	-	0	-	4	100	0	-	1	100	8↑	12
Gemmatimonadetes	0	-	0	-	0	-	1	0	0	-	0	-	0	
Nitrospirae	1	0	0	-	0	-	0	-	1↑	100	0	-	0	
Planctomycetes	1	0	0	-	0	-	0	-	0	-	0	-	0	
Proteobacteria	135↓	66	2	100	5	100	96↑	96	12	92	74↑	99	47	51
Spirochaetes	2	50	0	-	0	-	3	33	0	-	1	100	3	67
Thaumarchaeota	9↑	0	0	-	0	-	0	-	0	-	0	-	0	-
Verrucomicrobia	3	67	0	-	0	-	1	0	0	-	0	-	2	0

Residuals are according to Pearson Chi-squared test (P<0.001).

↑ Combinations with Pearson residuals >2.

↓ Combinations with Pearson residuals <−2.

The lack of a *nir* gene was particularly notable among species within the Bacteroidetes (42%), but also within the Deltaproteobacteria (71.4%), Firmicutes (27.5%), and Euryarchaeota (35.7%), with the frequencies of Z-type organisms being significantly higher than expected within these groups (Tables 1 and S2). The occurrence of S-type organisms was higher than expected within the Deinococcus-Thermus phylum. Ten genomes carried both a *nirK* and a *nirS* gene (Fig. 1; Table S1), including Gammaproteobacterium HdN1, *Rhodothermus marinus* and four strains of *Pseudomonas stutzeri* that also possessed a *nosZ* gene. Interestingly, the Bacteroidete *Rhodothermus marinus* did not have a *nor* gene although the other three genes were present.

### Gene co-occurrence patterns in relation to habitat preference

**Figure 3:**
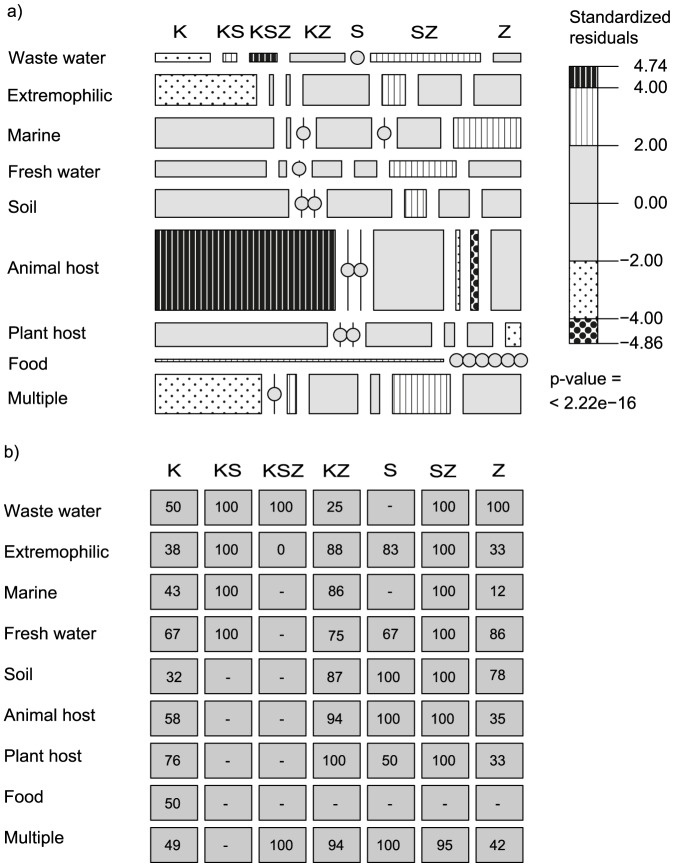
Gene co-occurrence types and habitat preference. a) Mosaic plot of *nirK* (K), *nirS* (S) and *nosZ* (Z) co-occurrence types across different habitat categories where tile size reflects the number of occurrences and patterns indicate significant overrepresentation (>2) or underrepresentation (<−2) of co-occurrence patterns, as determined by standardized Pearson residuals from χ^2^ test results (*P*<0.001). Circles indicate non-occurring combinations. b) The percentage of organisms harboring a *nor* gene for each combination across the habitat categories. No value indicates non-occurring combinations.

When comparing data relating to lifestyle in terms of habitat preference with *nir, nor* and *nosZ* gene co-occurrences, a significantly non-random pattern of distribution was observed across the different *nir* and *nos* categories (χ^2^ = 136, *P*<0.001; Fig. 3). Examination of standardized residuals revealed that K-type organisms were significantly overrepresented among animal host associated lifestyles, whereas the frequencies of both S- and SZ-types were significantly lower than expected in this category. By contrast, S-types were overrepresented among soil and extreme habitats, while K-types did not deviate from the expected frequency of occurrence or were underrepresented in each category, respectively. Contrasting patterns of K- and SZ-types were also observed in wastewater and multiple habitat categories, which had higher than expected frequencies of SZ-type organisms. Less than half (47%) of the K-type genomes did not have a *nor* gene and the percentage was especially low among organisms isolated from soil. However, in fresh water systems and among plant hosts 67 and 76%, respectively harbored *nor*. Interestingly, in wastewater the occurrence of organisms with both *nir* genes (KS) was significantly higher than expected, as well as organisms with a KSZ pattern. The frequency of organisms with only *nosZ* was overrepresented in marine ecosystems, yet lower than expected in the category of plant host-associated lifestyles. The Z-type organisms exhibited a low occurrence of *nor* in marine (12%) and host associated environments, whereas the opposite was observed in fresh water, soil and wastewater.

## Discussion

The genome data-set analyzed in this study demonstrated that the denitrification pathway is modular with no less than seven different co-occurrence types of the genes coding for the reductases NirK, NirS and NosZ. Interestingly, we found ten organisms that possess both a *nirK* and a *nirS* gene in the same genome. Although that is still rare, it contradicts the previous assumption that the two nitrite reductases are mutually exclusive [14]. The presence of homologues for both *nir* genes was previously reported in the genome of a *Methylomonas* strain [48]. This may be significant in light of recent findings by [49], who demonstrated the co-occurence of both *nir* genes in co-existing subpopulations affiliated to *Pseudovibrio* detected in the metagenome of a marine denitrifying community. Nevertheless, it has not yet been demonstrated if the two types of nitrite reductases are functional when present in the same genome, although it is not unlikely considering that two divergent copies of *nirS* in a *Thauera sp*. isolate were expressed under different conditions [50].

The *nosZ* gene was used as a proxy for the capacity of an organism to reduce N_2_O and we found that this gene occurred much more frequently in the genomes of organisms with *nirS* than in those with *nirK*. This was previously suggested [14], although the limited number of observations at that time did not support a significant pattern. The presence or absence of a *nor* gene partly explained this pattern, as *nirS* almost always co-occurred with *nor*, although there were exceptions. The substantial difference in co-occurrence of *nosZ* with *nirS* vs. *nirK* in the genome data-set is likely governed by an underlying ecological or evolutionary mechanism that constrains the loss of *nor* and *nosZ* in organisms with *nirS*. This is also supported by the higher degree of phylogenetic conservation observed for *nosZ* presence when mapped onto the *nirS* phylogeny than onto that of *nirK*. Previous studies have shown that loss of the *nos* regulon may occur through either short-term adaptive processes, such as phase variation in *nirK Azospirillum* soil isolates [51], or more long-term gene loss events as observed for *nirK Neisseria* species [52]. It is tempting to attribute the higher frequency of *nirS*, *nor* and *nosZ* co-occurrence to regulatory mechanisms that may be shared between the three enzyme complexes, but not with NirK, which is fully functional without accessory proteins. The expression of all three enzymes is controlled by oxygen and NO levels within the cell [11], [53] and initiated by the transcription factors NNR, NnrR or DNR [54], however no specific link between *nirS*, *nor* and *nosZ* expression has been described to date. Based on the gene co-occurrence patterns, the majority of *nirS* organisms are more likely to perform complete denitrification to N_2_ than the *nirK* types under favorable environmental conditions and we suggest that the presence of *nor* in the genome of an organism also harboring *nir* or *nosZ* serve as an indicator of whether the organism is a denitrifier *sensu stricto* or merely a nitrite or nitrous oxide reducer.

In the context of different co-occurrence patterns with *nosZ*, it is interesting that a range of studies have shown evidence of niche differentiation between *nirS* and *nirK* denitrifying communities [17]–[19], [21], [55]. If the co-occurrence patterns found in our genome comparisons are reflected in nature, an ecosystem in which the denitrifier community is dominated by *nirK* bearing organisms would be more likely to emit more N_2_O than one dominated by denitrifiers with *nirS* under the same environmental conditions. Indeed, the ratio of *nirS*/*nirK* type denitrifiers was recently demonstrated to have a significant and negative relationship with the capacity of a soil to act as an N_2_O sink when modeling the relative influence of abiotic and denitrifier community factors on the potential for soil N_2_O reduction [13]. This is also supported by previous findings. Clark et al. [56] reported a positive correlation between *nirK* abundance and N_2_O emissions from arable and woodland soils, and based on qPCR measurements estimated that only 10–30% of *nirK* type denitrifiers also carry the *nosZ* gene, depending on soil type. Similarly, Cuhel et al. [57] described a negative relationship between the ratio of N_2_O emissions to total denitrification activity (i.e. N_2_ + N_2_O) and the relative abundance of *nirS-*type denitrifiers in pastureland soils.

A highly truncated version of the denitrification pathway was observed among the substantial fraction of organisms that possessed the *nosZ* gene without having *nir* or a putatively N_2_O producing *nor* gene, and these were predominantly found within *nosZ* clade II. Several representatives from this clade, including the host-associated bacteria *Wolinella succinogenes* and *Campylobacter fetus*, as well as the thermophile *Geobacillus thermodenitrificans* and the soil bacterium *Anaeromyxobacter dehalogenans*, have been demonstrated to grow with N_2_O as the single electron acceptor [10], [12], [58]–[60]. An alternative, dissimilatory function for the nitrous oxide reductase has been recently proposed [61], but from an ecosystem perspective this detoxifying instead of energy conserving reaction would still result in N_2_O reduction. Net negative N_2_O fluxes in field measurements have been reported [62], [63] and recently it was demonstrated that both the abundance and phylogenetic diversity of *nosZ* clade II largely explained the soil N_2_O sink capacity [13]. The authors also showed that groups that were identified as significant indicators of N_2_O reduction were predominant in clade II communities, especially those associated with *nosZ* II lineages from organisms lacking *nir*. Because clade II has been shown to be both diverse and abundant in different ecosystems [45], [46], these organisms in particular can potentially be significant N_2_O consumers in the environment.

Although denitrification as a trait is widely distributed across different phyla, the observed *nir*/*nos* co-occurrence patterns were not randomly distributed across taxa, from phylum down to order level, with significant patterns related to certain taxonomic groups. The data set was too small to detect any patterns at lower taxonomic ranks. Accordingly, closely related organisms do not necessarily share this trait, and even if they do the denitrification genes may be distantly related or they may not have the same set of genes [8], [14], [64]–[66]. Organisms harboring a *nosZ* gene without possessing any *nir* gene were mainly found amongst the Bacteroidetes, and Firmicutes belonging to *nosZ* clade II, and predominantly lacked a *nor* gene. Thus, environments in which these taxa are highly abundant may have substantially lower N_2_O emission rates than those dominated by organisms that only have *nir* and *nor* genes, such as several representatives among the Actinobacteria and Ascomycota. This coincides with the results of Philippot et al [67], [68],who investigated the abundance of different taxa at high taxonomic ranks, as well as the abundance of different denitrifier genes and potential N_2_O production and denitrification rates on a field that was subjected to different cattle grazing regimes. Interestingly, the percentage of N_2_O to total denitrification activity (N_2_ + N_2_O) was lowest in the region of the field with the highest relative abundance of Bacteroidetes and lowest abundance of Actinobacteria. Furthermore, regions of the field in which *nirS* and *nosZ* clade I were most abundant also had the highest relative abundance of Betaproteobacteria, corresponding to our finding that the co-occurrence of *nirS* and *nosZ* is the predominant pattern of denitrification genes within this class. Thus, general patterns of gene co-occurrences in conjunction with taxonomic information can potentially aid in our interpretation of experimental data and predictions of genetic N_2_O emission potential.

Comparisons of gene co-occurrence patterns to preferred habitat categories showed differential associations of denitrifier gene combinations with specific habitats or lifestyles. Most notable was the prevalence of *K*-types over *S*-types amongst animal host associated organisms, suggesting that *nirK* might be involved to a greater extent during animal host adaptation and pathogenicity. Previous studies examining the relationship between nitrite reduction and pathogenicity of *Neisseria* and *Brucella* species have indicated that *nirK* is expressed primarily to help the bacterium cope with low oxygen levels within the host [69], [70]. However, *nirS* has also been shown to be important for virulence in *Pseudomonas aeruginosa*, albeit by regulation of virulence factors through maintaining a steady state of NO within the host [71]. It is known that prokaryotes undergo a process of genome shrinkage and gene loss during adaptation to animal hosts [72]. Since the *nirK* gene alone is sufficient to encode a functional protein while *nirS* requires several accessory genes [73], it may be more advantageous for host associated organisms to possess *nirK* rather than *nirS*. Interestingly, both the KS- and KSZ- types were significantly overrepresented in wastewater. One could speculate that carrying both types of *nir* genes could imply an adaptive advantage in an environment supporting denitrification activity, provided that both genes are transcribed to functional nitrite reductases under different conditions. The SZ type was more often found among genomes isolated from wastewater and freshwater than expected by chance. In agreement, it has been suggested that *nirS* denitrifiers are better adapted to stable, high water content conditions where oxygen availability is expected to be consistently low, while *nirK* communities tend to dominate under more recurrently changing conditions [74]. Other studies have also indicated that *nirS* communities are more frequently detected, more diverse and more phylogenetically clustered in marine systems than *nirK* communities [22], suggesting that *nirS* denitrifiers dominate in this habitat and that *nirS* denitrifying communities are shaped by habitat filtering more so than *nirK* communities. However, our analysis based on habitat data recovered from the GOLD database does not provide enough data or detail to confirm these hypotheses.

In conclusion, our results show that the co-occurrences of denitrification genes are not randomly distributed among taxonomic groups, preferred habitats or *nirK* and *nirS* denitrifiers. Although N_2_O emissions are subject to environmental factors and inherent cellular regulatory mechanisms, the ultimate control over whether N_2_O is emitted during denitrification is the presence of *nosZ* in the genomes of organisms that comprise the denitrifying community. Thus, the substantial difference between taxa as well as *nirS*, *nirK* and *nor* co-occurrence with *nosZ* likely plays a significant role in determining the genetic N_2_O emission potential from a given ecosystem, thereby underpinning the significance of microbial community structure for N_2_O emissions. Further research examining the underlying physiological or evolutionary mechanisms that result in the non-random patterns of *nir*/*nor*/*nos* gene occurrence observed here may provide additional predictive value for future N_2_O mitigation strategies.

## Supporting Information

Figure S1 **Maximum likelihood phylogeny of full-length 16S and 18S rRNA sequences from 652 organisms harboring denitrification genes.** The inner colored ring represents taxonomic affiliation as indicated by the legend. The four outer bar-chart rings show the presence of *nirK* (orange), *nirS* (purple), *nor* (turquoise) and *nosZ* (magenta). Bar height represents the number of copies (≤4). Bootstrap values >70% are indicated by grey circles, and the scale bar denotes nucleotide substitution rate (GTR+Γ).
(EPS)

Figure S2 **Maximum likelihood phylogeny of 458 full-length** ***nirK*** **amino acid sequences, rooted at midpoint.** Co-occurrences of *nirS*, *nor* and *nosZ* genes in the genomes are indicated with purple, turquoise and magenta bars, respectively. Bar height depicts the number of co-occurring gene copies (≤3) and the scale bar denotes nucleotide substitution rate (LG+Γ). Strain names are colored to indicate taxonomic affiliation according to the legend, and bootstrap values >70% are designated by circles.
(EPS)

Figure S3 **Maximum likelihood phylogeny of 110 full-length** ***nirS*** **amino acid sequences, rooted at midpoint.** Co-occurrences of *nirK*, *nor* and *nosZ* genes in the genomes are indicated with orange, turquoise and magenta bars, respectively. Bar height depicts the number of co-occurring gene copies (≤2) and the scale bar denotes nucleotide substitution rate (LG+Γ+F). Strain names are colored to indicate taxonomic affiliation according to the legend, and bootstrap values >70% are designated by circles.
(EPS)

Figure S4 **Maximum likelihood phylogenies of 413 prokaryotic and 18 fungal (inserted graph) full-length** ***nor*** **amino acid sequences**. Both phylogenies are rooted at midpoint. Co-occurrences of *nirK*, *nirS* and *nosZ* genes in the genomes are indicated with orange, purple and magenta bars, respectively. Bar height depicts the number of co-occurring gene copies (≤4) and the scale bar denotes nucleotide substitution rate (LG+Γ+F and WAG+Γ+F respectively). *cnorB* sequences are shaded in orange and *qnorB* sequences in green. Strain names are colored to indicate taxonomic affiliation according to the legend, and bootstrap values >70% are designated by circles.
(EPS)

Figure S5 **Maximum likelihood phylogeny of 314 full-length** ***nosZ*** **amino acid sequences, rooted at midpoint.** Co-occurrences of *nirK*, *nirS* and *nor* genes in the genomes are indicated with orange, purple and turquoise bars, respectively. Bar height depicts the number of co-occurring gene copies (≤4) and the scale bar denotes nucleotide substitution rate (LG+Γ). Clade I is shaded in orange, clade II in blue and the halophilic Archaea in turquoise. Strain names are colored to indicate taxonomic affiliation according to the legend, and bootstrap values >70% are designated by circles.
(EPS)

Table S1 **Taxon identification number and project name according to NCBI of 652 organisms harboring denitrification genes. Copy numbers of** ***nirK*** **,** ***nirS*** **,** ***nor*** **and** ***nosZ*** **are indicated.**
(PDF)

Table S2 **Frequency table of gene co-occurrence patterns and taxonomic affiliation at class level.** The *nirK* (K), *nirS* (S) and *nosZ* (Z) co-occurrence types and percentage of organisms within each class that also harbor a *nor* gene are indicated.
(PDF)

Table S3 **Frequency table of gene co-occurrence patterns and taxonomic affiliation at order level.** The *nirK* (K), *nirS* (S) and *nosZ* (Z) co-occurrence types and percentage of organisms within each order that also harbor a *nor* gene are indicated.
(PDF)
