# Supplementary material for: Intergenomic Comparisons Highlight Modularity of the Denitrification Pathway and Underpin the Importance of Community Structure for N2O Emissions
Source: PLoS One. 2014 Dec 1;9(12):e114118. doi: 10.1371/journal.pone.0114118 (PMC4250227; doi:10.1371/journal.pone.0114118)
Supplement: Table S1 — Taxon identification number and project name according to NCBI of 652 organisms harboring denitrification genes. Copy numbers of nirK , nirS , nor and nosZ are indicated. (PDF) [file pone.0114118.s006.pdf]

Table S1. Taxon identification number and project name according to NCBI of 652 organisms harboring denitrification genes. Copy numbers of *nirK*, *nirS*, *nor* and *nosZ* are indicated.

NCBI.TAXON.ID	NCBI.PROJECT.NAME	*nirK*	*nirS*	*nor*	*nosZ*
**477184**	Achromobacter arsenitoxydans SY8	1	0	1	1
**762376**	Achromobacter xylosoxidans A8	1	0	1	1
**1003200**	Achromobacter xylosoxidans AXX-A	1	0	1	1
**562971**	Achromobacter xylosoxidans C54	1	0	1	1
**351607**	Acidothermus cellulolyticus 11B	1	0	0	0
**573060**	Acidovorax delafieldii 2AN	0	1	2	1
**535289**	Acidovorax ebreus TPSY	0	1	2	1
**999386**	Acidovorax radicis N35	0	1	2	1
**232721**	Acidovorax sp. JS42	0	1	2	1
**512030**	Acidovorax sp. NO-1	0	0	1	1
**591023**	Actinobacillus minor 202	1	0	0	0
**416269**	Actinobacillus pleuropneumoniae L20	1	0	0	0
**434271**	Actinobacillus pleuropneumoniae serovar 3 str. JL03	1	0	0	0
**339671**	Actinobacillus succinogenes 130Z	1	0	1	0
**696748**	Actinobacillus suis H91-0380	1	0	0	0
**887324**	Actinobacillus ureae ATCC 25976	1	0	0	0
**525245**	Actinomyces coleocanis DSM 15436	1	0	0	0
**1125717**	Actinomyces georgiae F0490	1	0	0	0
**435830**	Actinomyces graevenitzii C83	1	0	0	0
**1125718**	Actinomyces massiliensis F0489	1	0	1	0
**1115803**	Actinomyces naeslundii 'Howell 279'	1	0	1	0
**411466**	Actinomyces odontolyticus ATCC 17982	1	0	0	0
**649742**	Actinomyces odontolyticus F0309	1	0	0	0
**871541**	Actinomyces oris K20	1	0	1	0
**521393**	Actinomyces sp. 7400942	1	0	1	0
**762963**	Actinomyces sp. oral taxon 170 str. F0386	1	0	1	0
**706439**	Actinomyces sp. oral taxon 171 str. F0337	1	0	1	0
**944560**	Actinomyces sp. oral taxon 175 str. F0384	1	0	1	0
**888051**	Actinomyces sp. oral taxon 178 str. F0338	1	0	0	0
**888052**	Actinomyces sp. oral taxon 180 str. F0310	1	0	0	0
**888056**	Actinomyces sp. oral taxon 448 str. F0400	1	0	1	0
**649743**	Actinomyces sp. oral taxon 848 str. F0332	1	0	0	0
**653386**	Actinomyces sp. oral taxon 849 str. F0330	1	0	1	0
**1118058**	Actinomyces sp. ph3	1	0	0	0
**1105029**	Actinomyces sp. str. ICM39	1	0	0	0
**936548**	Actinomyces sp. str. ICM47	1	0	0	0
**525246**	Actinomyces urogenitalis DSM 15434	1	0	0	0
**562973**	Actinomyces viscosus C505	1	0	1	0
**512565**	Actinoplanes missouriensis NBRC 102363	1	0	0	0
**446462**	Actinosynnema mirum DSM 43827	1	0	0	0
**746697**	Aequorivita sublithincola DSM 14238	1	0	0	1

**1208104**	Aeromonas media WS	0	0	0	1
**1197906**	Afipia birgiae 34632	0	0	0	1
**883080**	Afipia felis ATCC 53690	1	0	0	0
**666684**	Afipia sp. 1NLS2	3	0	1	1
**176299**	Agrobacterium tumefaciens str. C58	1	0	1	0
**447093**	Ajellomyces capsulatus G186AR	1	0	1	0
**946394**	Ajellomyces dermatitidis ATCC 18187	1	0	1	0
**1156919**	Alcaligenes faecalis Aunclassified	1	0	1	0
**1156918**	Alcaligenes faecalis subsp. faecalis NCIB 8687	1	0	1	1
**1069631**	Alcaligenes sp. HPC1271	1	0	1	1
**930169**	Alcanivorax dieselolei B5	1	0	1	1
**596153**	Alicycliphilus denitrificans BC	0	1	1	1
**596154**	Alicycliphilus denitrificans K601	0	1	1	1
**1200791**	Alicycliphilus sp. CRZ1	1	1	2	0
**1200346**	Alicyclobacillus hesperidum URH17-3-68	1	0	0	0
**187272**	Alkalilimnicola ehrlichei MLHE-1	0	0	1	1
**926569**	Anaerolinea thermophila UNI-1	0	1	0	0
**455488**	Anaeromyxobacter dehalogenans 2CP-1	0	0	2	1
**290397**	Anaeromyxobacter dehalogenans 2CP-C	0	0	3	1
**404589**	Anaeromyxobacter sp. Fw109-5	0	0	3	1
**447217**	Anaeromyxobacter sp. K	0	0	3	1
**76114**	Aromatoleum aromaticum EbN1	0	1	1	1
**861360**	Arthrobacter arilaitensis Re117 RE117	1	0	0	0
**663331**	Arthroderma benhamiae CBS 112371	1	0	1	0
**535722**	Arthroderma gypseum CBS 118893	1	0	1	0
**554155**	Arthroderma otae CBS 113480	1	0	0	0
**332952**	Aspergillus flavus NRRL3357	1	0	1	0
**330879**	Aspergillus fumigatus Af293	2	0	0	0
**510516**	Aspergillus oryzae RIB40	1	0	1	0
**927772**	Aspergillus sojae NBRC 4239	1	0	1	0
**341663**	Aspergillus terreus NIH2624	1	0	1	0
**1105030**	Atopobium sp. ICM58	1	0	0	0
**1184607**	Austwickia chelonae NBRC 105200	1	0	1	0
**1001584**	Avibacterium paragallinarum AVPAR72	1	0	0	0
**62928**	Azoarcus sp. BH72	0	0	1	1
**1003237**	Azospirillum amazonense Y2	0	0	0	1
**1064539**	Azospirillum brasilense	1	0	1	1
**862719**	Azospirillum lipoferum 4B	1	0	1	1
**137722**	Azospirillum sp. B510	1	0	1	0
**1131731**	Bacillus azotoformans LMG 9581	1	0	2	3
**1196031**	Bacillus oceanisediminis 2691	1	0	0	0
**1033734**	Bacillus sp. 10403023	1	0	0	0
**665959**	Bacillus sp. 2_A_57_CT2	1	0	0	0
**660997**	Bacillus sp. JC63	0	0	0	1
**264462**	Bdellovibrio bacteriovorus HD100	1	0	1	0
**1069642**	Bdellovibrio bacteriovorus str. Tiberius	1	0	1	0

**232259**	Belliella baltica DSM 15883	1	0	0	1
**1147128**	Bifidobacterium asteroides PRL2011	1	0	0	0
**1046627**	Bizionia argentinensis JUB59	1	0	1	1
**138336**	Blastococcus saxobsidens DD2	1	0	0	0
**340100**	Bordetella petrii DSM 12804	0	1	2	1
**446465**	Brachybacterium faecium DSM 4810	1	0	1	0
**1064537**	Brachybacterium paraconglomeratum LC44	1	0	0	0
**1074488**	Brachybacterium squillarum M-6-3	1	0	0	0
**1128253**	Bradyrhizobium japonicum CCBAU 15354	1	0	1	0
**1128255**	Bradyrhizobium japonicum CCBAU 15618	1	0	1	0
**224911**	Bradyrhizobium japonicum USDA 110	1	0	1	1
**1037409**	Bradyrhizobium japonicum USDA 6	1	0	2	0
**1128287**	Bradyrhizobium liaoningense CCBAU 05525	1	0	0	0
**288000**	Bradyrhizobium sp. BTAi1	1	0	1	1
**1128179**	Bradyrhizobium sp. CCBAU 15615	1	0	1	1
**1128180**	Bradyrhizobium sp. CCBAU 15635	1	0	1	1
**1128178**	Bradyrhizobium sp. CCBAU 43298	1	0	1	1
**1223566**	Bradyrhizobium sp. CCGE-LA001	1	0	1	0
**566679**	Bradyrhizobium sp. ORS 375	2	0	1	1
**114615**	Bradyrhizobium sp. ORS278	1	0	1	0
**115808**	Bradyrhizobium sp. ORS285	1	0	1	1
**335659**	Bradyrhizobium sp. S23321	1	0	1	0
**551936**	Bradyrhizobium sp. STM 3809	1	0	1	1
**551947**	Bradyrhizobium sp. STM 3843	1	0	1	1
**319003**	Bradyrhizobium sp. WSM1253	1	0	1	0
**319017**	Bradyrhizobium sp. WSM471	1	0	1	0
**1144344**	Bradyrhizobium sp. YR681	1	0	0	0
**1128326**	Bradyrhizobium yuanmingense CCBAU 05623	1	0	1	1
**1128327**	Bradyrhizobium yuanmingense CCBAU 25021	1	0	1	0
**391600**	Brevundimonas sp. BAL3	1	0	0	0
**1104320**	Brucella abortus A13334	1	0	1	1
**483179**	Brucella canis ATCC 23365	1	0	1	1
**595497**	Brucella ceti str. Cudo	1	0	1	1
**1148757**	Brucella melitensis S66	1	0	1	1
**568815**	Brucella microti CCM 4915	1	0	1	1
**520456**	Brucella neotomae 5K33	0	0	1	1
**444178**	Brucella ovis ATCC 25840	1	0	1	1
**520461**	Brucella pinnipedialis B2/94	1	0	1	1
**520449**	Brucella sp. 83/13	1	0	1	1
**470735**	Brucella sp. BO1	1	0	1	1
**204722**	Brucella suis 1330	1	0	1	1
**520488**	Brucella suis bv. 4 str. 40	1	0	1	1
**243160**	Burkholderia mallei ATCC 23344	2	0	2	1
**412022**	Burkholderia mallei NCTC 10229	2	0	2	0
**441162**	Burkholderia oklahomensis C6786	1	0	1	0
**441163**	Burkholderia oklahomensis EO147	1	0	1	0

**884204**	Burkholderia pseudomallei 1026b	2	0	2	1
**1086032**	Burkholderia pseudomallei 1258a	1	0	1	1
**441155**	Burkholderia pseudomallei B7210	2	0	2	1
**271848**	Burkholderia thailandensis E264	2	0	2	1
**441157**	Burkholderia thailandensis MSMB43	1	0	1	0
**864051**	Burkholderiales bacterium JOSHI_001	0	1	1	0
**986075**	Caldalkalibacillus thermarum TA2.A1	1	0	0	0
**926550**	Caldilinea aerophila DSM 14535 = NBRC 104270	0	0	0	1
**360104**	Campylobacter concisus 13826	0	0	1	1
**360105**	Campylobacter curvus 525.92	0	0	1	1
**360106**	Campylobacter fetus subsp. fetus 82-40	0	0	0	1
**983328**	Campylobacter fetus subsp. venerealis str. NCTC 10354	0	0	0	1
**553220**	Campylobacter gracilis RM3268	0	0	1	1
**553218**	Campylobacter rectus RM3267	0	0	0	1
**1244083**	Campylobacter showae CSUNSWCD	0	0	1	1
**553219**	Campylobacter showae RM3277	0	0	1	1
**665939**	Campylobacter sp. 10_1_50	0	0	1	1
**522306**	Candidatus Accumulibacter phosphatis clade IIA str. UW-1	0	1	1	1
**671143**	Candidatus Methylomirabilis oxyfera	0	1	3	0
**1231054**	Candidatus Microthrix parvicella Bio17-1	1	0	0	0
**886738**	Candidatus Nitrosoarchaeum limnia SFB1	1	0	0	0
**1229908**	Candidatus Nitrosopumilus koreensis AR1	1	0	0	0
**1229909**	Candidatus Nitrosopumilus sp. AR2	1	0	0	0
**859192**	Candidatus Nitrosopumilus sp. BG20	1	0	0	0
**497727**	Candidatus Nitrososphaera gargensis Ga9.2	2	0	0	0
**330214**	Candidatus Nitrospira defluvii	2	0	0	0
**553178**	Capnocytophaga gingivalis ATCC 33624	1	0	0	1
**936370**	Capnocytophaga sp. CM59	1	0	1	1
**706436**	Capnocytophaga sp. oral taxon 329 str. F0087	1	0	1	0
**888059**	Capnocytophaga sp. oral taxon 338 str. F0234	1	0	0	1
**553177**	Capnocytophaga sputigena ATCC 33612	1	0	1	0
**638300**	Cardiobacterium hominis ATCC 15826	1	0	1	1
**797473**	Cardiobacterium valvarum F0432	1	0	1	1
**509190**	Caulobacter segnis ATCC 21756	1	0	1	0
**1225176**	Cecembia lonarensis LW9	1	0	2	1
**1208323**	Celeribacter baekdonensis B30	1	0	1	1
**590998**	Cellulomonas fimi ATCC 484	1	0	0	0
**446466**	Cellulomonas flavigena DSM 20109	1	0	0	0
**688270**	Cellulophaga algicola DSM 14237	0	0	1	1
**593907**	Cellvibrio gilvus ATCC 13127	1	0	0	0
**306901**	Chaetomium globosum CBS 148.51	1	0	1	0
**759272**	Chaetomium thermophilum var. thermophilum DSM 1495	1	0	1	0
**1211115**	Chelatococcus sp. GW1	1	0	1	1
**326427**	Chloroflexus aggregans DSM 9485	1	0	0	0
**324602**	Chloroflexus aurantiacus J-10-fl	1	0	0	0
**480224**	Chloroflexus sp. Y-400-fl	1	0	0	0

**243365**	Chromobacterium violaceum ATCC 12472	1	0	1	0
**525257**	Chryseobacterium gleum ATCC 35910	1	0	1	0
**497964**	Chthoniobacter flavus Ellin428	1	0	0	0
**766499**	Citreicella sp. 357	0	0	0	1
**396776**	Coccidioides immitis H538.4	1	0	0	0
**469472**	Coccidioides posadasii CPA 0001	1	0	0	0
**490068**	Coccidioides posadasii CPA 0020	1	0	0	0
**490066**	Coccidioides posadasii RMSCC 1038	1	0	0	0
**443226**	Coccidioides posadasii str. Silveira	1	0	0	0
**167879**	Colwellia psychrerythraea 34H	0	1	1	1
**525260**	Corynebacterium accolens ATCC 49725	1	0	1	0
**548476**	Corynebacterium aurimucosum ATCC 700975	1	0	0	0
**698966**	Corynebacterium diphtheriae 241	1	0	1	0
**196164**	Corynebacterium efficiens YS-314	1	0	0	0
**645127**	Corynebacterium kroppenstedtii DSM 44385	1	0	0	0
**525264**	Corynebacterium pseudogenitalium ATCC 33035	1	0	0	0
**1074485**	Corynebacterium pseudotuberculosis 316	1	0	1	0
**1087453**	Corynebacterium pseudotuberculosis 42/02-A	1	0	1	0
**525268**	Corynebacterium striatum ATCC 6940	1	0	0	0
**553206**	Corynebacterium tuberculostearicum SK141	1	0	0	0
**504474**	Corynebacterium urealyticum DSM 7109	1	0	0	0
**1200775**	Cupriavidus basilensis B-8	0	1	1	1
**1217718**	Cupriavidus sp. BIS7	0	2	1	0
**164546**	Cupriavidus taiwanensis	0	1	0	0
**159087**	Dechloromonas aromatica RCB	0	2	1	2
**640081**	Dechlorosoma suillum PS	0	2	1	2
**522772**	Denitrovibrio acetiphilus DSM 12809	0	0	0	1
**756499**	Desulfitobacterium dehalogenans ATCC 51507	0	0	1	1
**871963**	Desulfitobacterium dichloroeliminans LMG P-21439	0	0	1	1
**537010**	Desulfitobacterium hafniense DP7	0	0	1	1
**138119**	Desulfitobacterium hafniense Y51	0	0	1	1
**706587**	Desulfomonile tiedjei DSM 6799	0	0	1	1
**768704**	Desulfosporosinus meridiei DSM 13257	0	0	1	1
**768710**	Desulfosporosinus youngiae DSM 17734	0	0	1	1
**696281**	Desulfotomaculum ruminis DSM 2154	0	0	0	1
**1077144**	Dietzia alimentaria 72	1	0	0	0
**910954**	Dietzia cinnamea P4	1	0	0	0
**398580**	Dinoroseobacter shibae DFL 12	0	1	1	1
**471854**	Dyadobacter fermentans DSM 18053	0	0	0	1
**1217721**	Dyella japonica A8	1	0	0	0
**1049564**	endosymbiont of Tevnia jerichonana	0	1	1	1
**589924**	Ferroglobus placidus DSM 10642	0	0	1	1
**531844**	Flavobacteriaceae bacterium 3519-10	1	0	0	1
**1178825**	Flavobacteriaceae bacterium P7-3-5	0	0	0	1
**391603**	Flavobacteriales bacterium ALC-1	0	0	0	1
**1041826**	Flavobacterium columnare ATCC 49512	1	0	1	1

**376686**	Flavobacterium johnsoniae UW101	1	0	1	0
**1144313**	Flavobacterium sp. CF136	1	0	1	0
**1202532**	Flavobacterium sp. F52	1	0	1	1
**643867**	Flexibacter tractuosus DSM 4126	1	0	1	1
**1215603**	Fusarium fujikuroi B14	1	0	1	0
**426428**	Fusarium oxysporum f. sp. lycopersici 4286 FGSC 4286	1	0	2	0
**555500**	Galbibacter sp. ck-I2-15	1	0	1	0
**1005058**	Gallibacterium anatis UMN179	1	0	1	0
**83406**	gamma proteobacterium HdN1	1	1	4	1
**379066**	Gemmatimonas aurantiaca T-27	1	0	0	1
**1234664**	Geobacillus caldoxylosilyticus CIC9	1	0	1	1
**235909**	Geobacillus kaustophilus HTA426	1	0	1	0
**495036**	Geobacillus sp. G11MC16	1	0	1	1
**581103**	Geobacillus sp. Y4.1MC1	1	0	1	0
**420246**	Geobacillus thermodenitrificans NG80-2	1	0	1	1
**634956**	Geobacillus thermoglucosidasius C56-YS93	1	0	1	0
**1136178**	Geobacillus thermoglucosidasius GT20	1	0	1	0
**334819**	Gibberella moniliformis 7600	1	0	2	0
**865937**	Gillisia limnaea DSM 15749	1	0	0	1
**493475**	Glaciecola arctica BSs20135	1	0	1	0
**411154**	Gramella forsetii KT0803	0	0	0	1
**1095744**	Haemophilus parahaemolyticus HK385	1	0	0	0
**888828**	Haemophilus parainfluenzae ATCC 33392	1	0	1	0
**1095746**	Haemophilus parainfluenzae HK2019	1	0	1	0
**1095745**	Haemophilus parainfluenzae HK262	1	0	1	0
**862965**	Haemophilus parainfluenzae T3T1	1	0	1	0
**1095743**	Haemophilus paraphrohaemolyticus HK411	1	0	0	0
**128398**	Haemophilus pittmaniae HK 85	1	0	0	0
**349521**	Hahella chejuensis KCTC 2396	0	1	2	1
**760192**	Haliscomenobacter hydrossis DSM 1100	0	0	1	1
**634497**	Haloarcula hispanica ATCC 33960	1	0	1	1
**272569**	Haloarcula marismortui ATCC 43049	1	0	1	1
**751944**	Halobacterium sp. DL1	0	0	0	1
**523841**	Haloferax mediterranei ATCC 33500	0	0	1	1
**309800**	Haloferax volcanii DS2	1	0	1	0
**469382**	Halogeometricum borinquense DSM 11551	1	0	1	1
**485914**	Halomicrobium mukohataei DSM 12286	1	0	1	0
**590061**	Halomonas sp. KM-1	0	1	1	1
**756883**	halophilic archaeon DL31	0	0	0	1
**797210**	Halopiger xanaduensis SH-6	2	0	2	1
**519442**	Halorhabdus utahensis DSM 12940	1	0	0	0
**416348**	Halorubrum lacusprofundi ATCC 49239	0	0	1	1
**543526**	Haloterrigena turkmenica DSM 5511	1	0	2	0
**864073**	Herbaspirillum frisingense GSF30	1	0	1	0
**204773**	Herminiimonas arsenicoxydans	1	0	2	0
**316274**	Herpetosiphon aurantiacus ATCC 23779	1	0	0	0

**1226784**	Histoplasma capsulatum var. capsulatum Tmu	1	0	1	0
**608538**	Hydrogenobacter thermophilus TK-6	0	1	1	1
**670307**	Hyphomicrobium denitrificans 1NES1	1	0	1	1
**582899**	Hyphomicrobium denitrificans ATCC 51888	1	0	1	1
**283942**	Idiomarina loihiensis L2TR	1	0	1	0
**945713**	Ignavibacterium album JCM 16511	0	0	0	1
**946077**	Imtechella halotolerans K1	1	0	1	1
**1189612**	Indibacter alkaliphilus LW1	1	0	2	0
**710696**	Intrasporangium calvum DSM 43043	1	0	1	0
**471856**	Jonesia denitrificans DSM 20603	1	0	0	0
**523791**	Kangiella koreensis DSM 16069	1	1	1	0
**1184609**	Kineosphaera limosa NBRC 100340	1	0	1	0
**888741**	Kingella denitrificans ATCC 33394	1	0	1	1
**1188007**	Kingella kingae 11220434	1	0	1	1
**887327**	Kingella kingae ATCC 23330	1	0	1	1
**1150867**	Kingella kingae PYKK081	1	0	1	1
**629741**	Kingella oralis ATCC 51147	1	0	1	1
**384765**	Labrenzia aggregata IAM 12614	0	1	1	1
**887898**	Lautropia mirabilis ATCC 51599	1	0	1	1
**929563**	Leptonema illini DSM 21528	0	1	1	1
**355278**	Leptospira biflexa serovar Patoc strain 'Patoc 1 (Ames)'	1	0	1	1
**1049789**	Leptospira broomii str. 5399	1	0	0	1
**1193011**	Leptospira fainei sv. Hurstbridge BUT 6T	1	0	0	1
**1049790**	Leptospira inadai sv. Lyme 10	0	0	0	1
**1049971**	Leptospira licerasiae str. MMD4847	0	0	1	1
**1193051**	Leptospira meyeri sv. Hardjo Went 5	1	0	1	0
**1193069**	Leptospira wolffii Khorat-H2	0	0	1	1
**395495**	Leptothrix cholodnii SP-6	0	1	1	1
**279714**	Lutiella nitroferrum 2002	0	1	1	1
**342108**	Magnetospirillum magneticum AMB-1	0	2	1	1
**272627**	Magnetospirillum magnetotacticum MS-1	0	1	0	1
**221988**	Mannheimia succiniciproducens MBEL55E	1	0	1	0
**313603**	Maribacter sp. HTCC2170	1	0	1	1
**859350**	Marine archaeal group 1 strain BD31	1	0	0	0
**857392**	Marinilabilia sp. AK2	1	0	2	1
**351348**	Marinobacter aquaeolei VT8	0	1	1	1
**1163748**	Marinobacter hydrocarbonoclasticus str. ATCC 49840	0	1	2	1
**490759**	Marinobacter sp. BSs20148	1	0	1	1
**270374**	Marinobacter sp. ELB17	1	0	1	1
**1094979**	Marinobacter sp. MnI7-9	0	0	0	1
**314271**	Maritimibacter alkaliphilus HTCC2654	1	0	1	1
**1191523**	Melioribacter roseus P3M	0	0	0	1
**1107882**	Mesorhizobium alhagi CCNWXJ12-2	2	0	0	0
**1082933**	Mesorhizobium amorphae CCNWGS0123	1	0	0	0
**754035**	Mesorhizobium australicum WSM2073	1	0	0	0
**765698**	Mesorhizobium ciceri biovar biserrulae WSM1271	1	0	0	0

**536019**	Mesorhizobium opportunistum WSM2075	1	0	0	0
**266779**	Mesorhizobium sp. BNC1	2	0	0	0
**1156937**	Methylacidiphilum fumariolicum SolV	2	0	1	0
**481448**	Methylacidiphilum infernorum V4	1	0	1	0
**426117**	Methylobacterium sp. 4-46	0	0	0	1
**395965**	Methylocella silvestris BL2	1	0	1	0
**622637**	Methylocystis sp. ATCC 49242	1	0	0	0
**754476**	Methylophaga sp. JAM1	0	0	2	1
**754477**	Methylophaga sp. JAM7	1	0	0	0
**583345**	Methylotenera mobilis JLW8	1	0	1	0
**1032480**	Microlunatus phosphovorus NM-1	1	0	1	0
**644283**	Micromonospora aurantiaca ATCC 27029	1	0	0	0
**648999**	Micromonospora sp. L5	1	0	0	0
**1089455**	Mobilicoccus pelagius NBRC 104925	1	0	1	0
**857581**	Moraxella catarrhalis 7169	1	0	1	0
**1201293**	Moritella dasanensis ArB 0140	0	0	0	1
**1202962**	Moritella marina ATCC 15381	0	0	0	1
**58051**	Moritella sp. PE36	0	0	0	1
**886377**	Muricauda ruestringensis DSM 13258	1	0	1	1
**1078013**	Mycobacterium avium subsp. avium Env 77	1	0	1	0
**1122247**	Mycobacterium hassiacum DSM 44199	1	0	0	0
**1232724**	Mycobacterium indicus pranii	1	0	2	0
**1138383**	Mycobacterium intracellulare MOTT-64	1	0	2	0
**875328**	Mycobacterium sp. JDM601	1	0	1	0
**278137**	Mycobacterium sp. Spyr1	1	0	0	0
**350058**	Mycobacterium vanbaalenii PYR-1	1	0	0	0
**1008457**	Myroides injenensis M09-0166	1	0	1	1
**883150**	Myroides odoratimimus CCUG 10230	1	0	1	0
**883154**	Myroides odoratimimus CIP 101113	1	0	1	1
**883155**	Myroides odoratimimus CIP 103059	1	0	1	1
**929704**	Myroides odoratus DSM 2801	1	0	1	1
**348780**	Natronomonas pharaonis DSM 2160	1	0	0	0
**888742**	Neisseria bacilliformis ATCC BAA-1200	1	0	1	0
**546262**	Neisseria cinerea ATCC 14685	1	0	1	1
**546263**	Neisseria elongata subsp. glycolytica ATCC 29315	1	0	1	0
**546264**	Neisseria flavescens NRL30031/H210	1	0	1	1
**596320**	Neisseria flavescens SK114	1	0	1	1
**489653**	Neisseria lactamica 020-06	1	0	1	1
**933280**	Neisseria lactamica NS19	1	0	1	1
**997348**	Neisseria macacae ATCC 33926	1	0	1	1
**1069618**	Neisseria meningitidis 92045	1	0	1	0
**1069616**	Neisseria meningitidis 98008	1	0	1	0
**630588**	Neisseria meningitidis alpha710	1	0	1	0
**1123520**	Neisseria meningitidis DE9686	1	0	1	0
**768485**	Neisseria meningitidis K1207	1	0	1	0
**122586**	Neisseria meningitidis MC58	1	0	1	0

**1079752**	Neisseria meningitidis Nm3127	1	0	1	0
**935589**	Neisseria meningitidis NZ-05/33	1	0	1	0
**546266**	Neisseria mucosa ATCC 25996	1	0	1	1
**435832**	Neisseria mucosa C102	1	0	1	1
**546267**	Neisseria polysaccharea ATCC 43768	1	0	1	1
**933266**	Neisseria sicca 4320	1	0	1	1
**547045**	Neisseria sicca ATCC 29256	1	0	1	1
**665946**	Neisseria sp. GT4A_CT1	1	0	1	0
**641149**	Neisseria sp. oral taxon 014 str. F0314	1	0	1	1
**546268**	Neisseria subflava NJ9703	1	0	1	1
**1051972**	Neisseria weaveri ATCC 51223	1	0	1	0
**1051985**	Neisseria weaveri LMG 5135	1	0	1	0
**331117**	Neosartorya fischeri NRRL 181	1	0	0	0
**929713**	Niabella soli DSM 19437	0	0	1	1
**700598**	Niastella koreensis GR20-10	0	0	0	1
**749222**	Nitratifractor salsuginis DSM 16511	0	1	1	1
**1189611**	Nitratireductor aquibiodomus RA22	1	0	0	0
**1231190**	Nitratireductor indicus C115	0	0	0	1
**391937**	Nitratireductor pacificus pht-3B	1	0	0	0
**387092**	Nitratiruptor sp. SB155-2	0	1	1	1
**323097**	Nitrobacter hamburgensis X14	1	0	0	0
**314253**	Nitrobacter sp. Nb-311A	1	0	0	0
**323098**	Nitrobacter winogradskyi Nb-255	1	0	0	0
**472759**	Nitrosococcus halophilus Nc4	1	0	1	0
**314279**	Nitrosococcus oceani AFC27	1	0	0	0
**323261**	Nitrosococcus oceani ATCC 19707	1	0	1	0
**105559**	Nitrosococcus watsoni C-113	1	0	2	0
**228410**	Nitrosomonas europaea ATCC 19718	1	0	1	0
**335283**	Nitrosomonas eutropha C91	1	0	1	0
**153948**	Nitrosomonas sp. AL212	1	0	1	0
**261292**	Nitrosomonas sp. Is79A3	1	0	0	0
**436308**	Nitrosopumilus maritimus SCM1	2	0	0	0
**1001994**	Nitrosopumilus sp. MY1	1	0	0	0
**323848**	Nitrosospira multiformis ATCC 25196	1	0	1	0
**1206733**	Nocardia niigatensis NBRC 100131	1	0	1	0
**196162**	Nocardioides sp. JS614	1	0	0	0
**511062**	Oceanimonas sp. GK1	1	0	1	0
**1231392**	Oceaniovalibus guishaninsula JLT2003	1	0	1	1
**670487**	Oceanithermus profundus DSM 14977	0	1	1	0
**439375**	Ochrobactrum anthropi ATCC 49188	2	0	1	1
**641118**	Ochrobactrum intermedium LMG 3301	1	0	1	0
**391626**	Octadecabacter antarcticus 307	1	0	0	0
**1031710**	Oligotropha carboxidovorans OM4	2	0	0	0
**504832**	Oligotropha carboxidovorans OM5	2	0	0	1
**278957**	Opitutaceae bacterium TAV2	0	0	0	1
**794903**	Opitutaceae bacterium TAV5	0	0	0	1

**452637**	Opitutus terrae PB90-1	1	0	0	1
**253245**	Owenweeksia hongkongensis DSM 17368	0	0	0	1
**621372**	Paenibacillus sp. oral taxon 786 str. D14	1	0	0	0
**482561**	Paracoccidioides brasiliensis Pb03	1	0	1	0
**318586**	Paracoccus denitrificans PD1222	0	1	1	1
**1051075**	Paracoccus denitrificans SD1	1	0	1	1
**412597**	Paracoccus sp. TRP	0	1	1	1
**864564**	Parascardovia denticolens DSM 10105	1	0	0	0
**641144**	Parascardovia denticolens F0305	1	0	0	0
**1193128**	Parascardovia denticolens IPLA 20019	1	0	0	0
**402881**	Parvibaculum lavamentivorans DS-1	1	0	1	0
**1095749**	Pasteurella bettyae CCUG 2042	1	0	0	0
**1206103**	Pedobacter arcticus A12	1	0	0	1
**151895**	Pedobacter saltans DSM 12145	0	0	0	1
**500485**	Penicillium chrysogenum Wisconsin 54-1255	1	0	0	0
**123214**	Persephonella marina EX-H1	0	1	1	1
**383629**	Phaeobacter gallaeciensis 2.10	1	0	1	0
**1002340**	Phaeobacter gallaeciensis ANG1	1	0	1	1
**391619**	Phaeobacter gallaeciensis DSM 17395	1	0	1	0
**450851**	Phenylobacterium zucineum HLK1	1	0	1	0
**1204719**	Phocaeicola abscessus CCUG 55929	0	0	0	1
**314280**	Photobacterium profundum 3TCK	0	0	1	1
**298386**	Photobacterium profundum SS9	0	0	1	1
**247490**	planctomycete KSU-1	1	0	0	0
**365044**	Polaromonas naphthalenivorans CJ2	1	0	1	0
**52972**	Polaromonas sp. CF318	1	0	0	0
**991905**	Polymorphum gilvum SL003B-26A1	0	1	1	1
**1144253**	Pontibacter sp. BAB1700	1	0	1	2
**944557**	Prevotella denticola CRIS 18C-A	0	0	0	1
**28129**	Prevotella denticola F0289	0	0	0	1
**857291**	Prevotella histicola F0411	0	0	0	1
**688246**	Prevotella multisaccharivorax DSM 17128	0	0	0	1
**702438**	Prevotella oulorum F0390	0	0	0	1
**1171373**	Propionibacterium acidipropionici ATCC 4875	1	0	2	0
**765117**	Propionibacterium acnes HL002PA3	1	0	1	0
**765122**	Propionibacterium acnes HL037PA1	1	0	1	0
**765101**	Propionibacterium acnes HL050PA2	1	0	1	0
**765084**	Propionibacterium acnes HL060PA1	1	0	1	0
**765114**	Propionibacterium acnes HL063PA2	1	0	1	0
**765074**	Propionibacterium acnes HL072PA1	1	0	1	0
**1051006**	Propionibacterium acnes SK182B-JCVI	1	0	1	0
**1114966**	Propionibacterium acnes TypeIA2 P.acn33	1	0	1	0
**997355**	Propionibacterium avidum ATCC 25577	1	0	0	0
**380773**	Propionibacterium freudenreichii subsp. shermanii CIP 103027	1	0	0	0
**767029**	Propionibacterium propionicum F0230a	1	0	0	0
**450748**	Propionibacterium sp. 5_U_42AFAA	1	0	1	0

**1005703**	Propionibacterium sp. CC003-HC2	1	0	1	0
**326442**	Pseudoalteromonas haloplanktis TAC125	1	0	1	0
**748280**	Pseudogulbenkiania sp. NH8B	0	1	1	1
**1163393**	Pseudomonas aeruginosa ATCC 25324	0	1	1	1
**1211113**	Pseudomonas aeruginosa DQ8	0	1	1	1
**557722**	Pseudomonas aeruginosa LESB58	0	1	1	1
**381754**	Pseudomonas aeruginosa PA7	0	2	1	1
**509633**	Pseudomonas aeruginosa PAb1	0	1	1	1
**994484**	Pseudomonas brassicacearum subsp. brassicacearum NFM421	0	1	2	2
**1037915**	Pseudomonas chlororaphis O6	1	0	1	0
**1038921**	Pseudomonas chlororaphis subsp. aureofaciens 30-84	1	0	0	0
**1117110**	Pseudomonas chlororaphis subsp. chlororaphis GP72	1	0	1	0
**384676**	Pseudomonas entomophila L48	1	0	0	0
**1117958**	Pseudomonas extremaustralis 14-3 substr. 14-3b	0	0	1	1
**1231390**	Pseudomonas fluorescens BS2	0	1	1	0
**1114970**	Pseudomonas fluorescens F113	0	1	2	2
**1221522**	Pseudomonas fluorescens NCIMB 11764	0	1	1	1
**564422**	Pseudomonas fluorescens NZ17	1	0	0	0
**220664**	Pseudomonas fluorescens Pf-5	1	0	0	0
**1038922**	Pseudomonas fluorescens Q2-87	0	1	1	1
**1038923**	Pseudomonas fluorescens Q8r1-96	0	1	1	1
**1007656**	Pseudomonas fluorescens Wayne1	2	0	0	0
**746360**	Pseudomonas fluorescens WH6	0	1	1	0
**1147786**	Pseudomonas mandelii JR-1	0	2	2	2
**1001585**	Pseudomonas mendocina NK-01	1	0	1	1
**665948**	Pseudomonas sp. 2_1_26	0	1	1	1
**440512**	Pseudomonas sp. Chol1	0	1	2	2
**1144323**	Pseudomonas sp. GM17	1	0	1	0
**1144708**	Pseudomonas sp. GM41(2012)	0	1	1	1
**1144334**	Pseudomonas sp. GM60	0	1	1	1
**1144338**	Pseudomonas sp. GM79	0	1	1	0
**1163398**	Pseudomonas sp. HYS	0	1	1	0
**1225174**	Pseudomonas sp. S5.2	1	0	1	1
**1226994**	Pseudomonas sp. TX1	1	0	1	0
**32042**	Pseudomonas stutzeri ATCC 14405 = CCUG 16156	0	1	2	1
**96563**	Pseudomonas stutzeri ATCC 17588 = LMG 11199	0	1	2	1
**1196835**	Pseudomonas stutzeri CCUG 29243	1	1	2	1
**1123519**	Pseudomonas stutzeri DSM 10701	1	1	2	1
**996285**	Pseudomonas stutzeri DSM 4166	0	1	1	1
**1218352**	Pseudomonas stutzeri KOS6	0	1	1	1
**1109445**	Pseudomonas stutzeri SDM-LAC	1	1	2	1
**1210443**	Pseudomonas stutzeri T13	1	1	2	1
**477228**	Pseudomonas stutzeri TS44	0	1	2	2
**911045**	Pseudovibrio sp. FO-BEG1	0	1	2	1
**1211114**	Pseudoxanthomonas sp. GW2	1	0	1	0
**743721**	Pseudoxanthomonas suwonensis strain 11-1	1	0	1	0

**1002339**	Psychrobacter sp. 1501(2011)	1	0	1	0
**313595**	Psychroflexus torquis ATCC 700755	0	0	0	1
**357804**	Psychromonas ingrahamii 37	0	0	0	1
**1007105**	Pusillimonas sp. T7-7	2	0	2	0
**178306**	Pyrobaculum aerophilum str. IM2	0	1	1	0
**340102**	Pyrobaculum arsenaticum DSM 13514	0	1	1	0
**410359**	Pyrobaculum calidifontis JCM 11548	0	1	1	1
**1104324**	Pyrobaculum sp. 1860	0	1	1	1
**381666**	Ralstonia eutropha H16	0	1	2	1
**264198**	Ralstonia eutropha JMP134	0	1	1	0
**1042878**	Ralstonia eutropha N-1	0	1	1	0
**266264**	Ralstonia metallidurans CH34	0	2	1	1
**428406**	Ralstonia pickettii 12D	1	0	1	1
**267608**	Ralstonia solanacearum GMI1000	1	0	1	1
**564065**	Ralstonia solanacearum MolK2	1	0	1	0
**859657**	Ralstonia solanacearum PSI07	1	0	1	0
**658080**	Ralstonia sp. 5_2_56FAA	1	0	1	1
**314283**	Reinekea sp. MED297	0	0	0	1
**538025**	Rhizobium etli 8C-3	1	0	0	0
**347834**	Rhizobium etli CFN 42	1	0	1	0
**526947**	Rhizobium etli GR56	1	0	0	0
**754764**	Rhizobium leguminosarum bv. trifolii WSM597	1	0	1	1
**754774**	Rhizobium leguminosarum bv. viciae USDA 2370	1	0	1	0
**1128399**	Rhizobium phaseoli Ch24-10	1	0	1	0
**1144312**	Rhizobium sp. CF122	1	0	1	0
**1144314**	Rhizobium sp. CF142	1	0	1	0
**394**	Rhizobium sp. NGR234	1	0	1	0
**1163408**	Rhodanobacter fulvus Jip2	3	0	3	1
**1162282**	Rhodanobacter sp. 115	4	0	2	1
**1076650**	Rhodanobacter sp. 116-2	2	0	0	1
**666685**	Rhodanobacter sp. 2APBS1	2	0	3	1
**1163407**	Rhodanobacter spathiphylli B39	1	0	2	1
**1163409**	Rhodanobacter thiooxydans LCS2	2	0	3	1
**272942**	Rhodobacter capsulatus SB1003	0	0	0	1
**371731**	Rhodobacter sp. SW2	0	1	1	0
**349102**	Rhodobacter sphaeroides ATCC 17025	1	0	1	1
**557760**	Rhodobacter sphaeroides KD131	1	0	1	1
**992186**	Rhodobacter sphaeroides WS8N	0	0	1	1
**439496**	Rhodobacterales bacterium Y4I	1	0	1	1
**450821**	Rhodococcus sp. P14	1	0	0	0
**338969**	Rhodoferax ferrireducens T118	0	0	1	1
**316055**	Rhodopseudomonas palustris BisA53	1	0	1	1
**316056**	Rhodopseudomonas palustris BisB18	0	0	0	1
**258594**	Rhodopseudomonas palustris CGA009	2	0	1	1
**652103**	Rhodopseudomonas palustris DX-1	1	0	1	1
**316058**	Rhodopseudomonas palustris HaA2	0	0	0	1

**395960**	Rhodopseudomonas palustris TIE-1	2	0	1	1
**414684**	Rhodospirillum centenum SW	0	0	1	1
**518766**	Rhodothermus marinus DSM 4252	1	1	0	1
**762570**	Rhodothermus marinus SG0.5JP17-172	1	0	0	1
**1187851**	Rhodovulum sp. PH10	1	0	0	0
**1228997**	Riemerella anatipestifer RA-CH-1	0	0	0	1
**992406**	Riemerella anatipestifer RA-GD	0	0	0	1
**313596**	Robiginitalea biformata HTCC2501	0	0	0	1
**744980**	Roseibium sp. TrichSKD4	1	0	0	0
**383372**	Roseiflexus castenholzii DSM 13941	0	1	0	0
**375451**	Roseobacter denitrificans OCh 114	0	1	1	1
**391595**	Roseobacter litoralis Och 149	0	1	1	1
**388739**	Roseobacter sp. SK209-2-6	1	0	1	1
**314264**	Roseovarius sp. 217	1	0	1	1
**391613**	Roseovarius sp. TM1035	0	0	0	1
**1125724**	Rothia aeria F0474	1	0	1	0
**762948**	Rothia dentocariosa ATCC 17931	1	0	1	0
**563032**	Rothia dentocariosa M567	1	0	1	0
**680646**	Rothia mucilaginosa	1	0	1	0
**553201**	Rothia mucilaginosa ATCC 25296	1	0	1	0
**563033**	Rothia mucilaginosa M508	1	0	1	0
**987059**	Rubrivivax benzoatilyticus JA2	0	1	1	0
**1154769**	Rubrivivax gelatinosus CBS	0	1	1	1
**983917**	Rubrivivax gelatinosus IL144	0	1	1	1
**246200**	Ruegeria pomeroyi DSS-3	0	1	1	1
**981384**	Ruegeria sp. TW15	0	1	1	1
**761193**	Runella slithyformis DSM 19594	0	0	0	1
**882083**	Saccharomonospora marina XMU15	1	0	0	0
**309807**	Salinibacter ruber DSM 13855	0	0	0	1
**146919**	Salinibacter ruber M8	0	0	0	1
**1033802**	Salinisphaera shabanensis E1L3A	1	0	1	1
**1095772**	Sanguibacter sp. JC301	1	0	0	0
**641146**	Scardovia inopinata F0304	1	0	0	0
**857290**	Scardovia wiggsiae F0424	2	0	0	0
**1043205**	Serinicoccus marinus MCCC 1A05965	1	0	0	0
**326297**	Shewanella amazonensis SB2B	1	0	1	0
**318161**	Shewanella denitrificans OS217	1	0	1	1
**323850**	Shewanella loihica PV-4	1	0	1	1
**392500**	Shewanella woodyi ATCC 51908	1	0	1	0
**580332**	Sideroxydans lithotrophicus ES-1	0	1	1	0
**644107**	Silicibacter lacuscaerulensis ITI-1157	0	1	1	1
**644076**	Silicibacter sp. TrichCH4B	1	0	1	0
**641147**	Simonsiella muelleri ATCC 29453	1	0	1	1
**1128329**	Sinorhizobium fredii CCBAU 05557	1	0	1	0
**1128330**	Sinorhizobium fredii CCBAU 25509	1	0	1	0
**1117943**	Sinorhizobium fredii HH103	1	0	1	0

**1185652**	Sinorhizobium fredii USDA 257	1	0	1	1
**366394**	Sinorhizobium medicae WSM419	1	0	1	0
**266834**	Sinorhizobium meliloti 1021	1	0	1	1
**794846**	Sinorhizobium sp. CCBAU 05631	1	0	1	0
**929556**	Solitalea canadensis DSM 3403	1	0	1	1
**479434**	Sphaerobacter thermophilus DSM 20745	1	0	0	1
**392499**	Sphingomonas wittichii RW1	1	0	2	0
**639283**	Starkeya novella DSM 506	1	0	1	0
**996637**	Streptomyces griseoaurantiacus M045	1	0	0	0
**1038929**	Streptomyces xinghaiensis S187	1	0	1	0
**479432**	Streptosporangium roseum DSM 43021	1	0	0	0
**679936**	Sulfobacillus acidophilus DSM 10332	1	0	0	0
**1051632**	Sulfobacillus acidophilus TPY	1	0	0	0
**1163617**	Sulfuricella denitrificans skB26	0	2	1	1
**563040**	Sulfurimonas autotrophica DSM 16294	0	1	2	1
**326298**	Sulfurimonas denitrificans DSM 1251	0	1	1	2
**929558**	Sulfurimonas sp. GD1	0	1	1	1
**387093**	Sulfurovum sp. NBC37-1	0	1	1	1
**292459**	Symbiobacterium thermophilum IAM 14863	1	0	0	0
**1008459**	Taylorella asinigenitalis MCE3	1	0	0	0
**743973**	Taylorella equigenitalis ATCC 35865	1	0	0	0
**937774**	Taylorella equigenitalis MCE9	1	0	0	0
**1123366**	Thalassospira xiamenensis M-5 DSM 17429	0	1	1	1
**1234381**	Thauera aminoaromatica S2	0	1	1	1
**1123367**	Thauera linaloolentis 47Lol = DSM 12138	0	1	1	2
**1234382**	Thauera phenylacetica B4P	0	2	1	1
**905052**	Thauera selenatis AX	0	1	1	1
**305700**	Thauera sp. 27	0	2	1	1
**303682**	Thauera sp. 28	0	2	1	1
**497321**	Thauera sp. 63	0	2	1	1
**85643**	Thauera sp. MZ1T	0	1	1	1
**644966**	Thermaerobacter marianensis DSM 12885	1	0	0	0
**867903**	Thermaerobacter subterraneus DSM 13965	1	0	0	0
**525904**	Thermobaculum terrenum ATCC BAA-798	1	0	0	0
**269800**	Thermobifida fusca YX	1	0	0	0
**469371**	Thermobispora bispora DSM 43833	1	0	0	0
**309801**	Thermomicrobium roseum DSM 5159	0	0	0	1
**751945**	Thermus oshimai JL-2	1	1	1	0
**743525**	Thermus scotoductus SA-01	1	1	1	0
**798128**	Thermus thermophilus JL-18	0	1	1	0
**762633**	Thermus thermophilus SG0.5JP17-16	0	1	1	0
**396595**	Thioalkalivibrio sp. K90mix	2	0	0	0
**713587**	Thioalkalivibrio thiocyanoxidans ARh 4	0	0	1	1
**292415**	Thiobacillus denitrificans ATCC 25259	0	1	2	1
**768671**	Thiocapsa marina	0	0	1	1
**559882**	Trichophyton equinum CBS127.97	1	0	0	0

**34387**	Trichophyton tonsurans CBS 112818	1	0	1	0
**663202**	Trichophyton verrucosum HKI 0517	1	0	1	0
**869212**	Turneriella parva DSM 21527	1	0	0	0
**336963**	Uncinocarpus reesii 1704	1	0	1	0
**1089683**	uncultured marine crenarchaeote 'Gulf of Maine'	3	0	0	0
**655186**	uncultured SUP05 cluster bacterium	1	0	1	0
**675816**	Vibrio orientalis CIP 102891	0	0	0	1
**1051646**	Vibrio tubiashii ATCC 19109	0	0	0	1
**866909**	Vibrio tubiashii NCIMB 1337	0	0	0	1
**1196028**	Virgibacillus halodenitrificans 1806	1	0	0	0
**273121**	Wolinella succinogenes DSM 1740	0	0	0	1
**446471**	Xylanimonas cellulosilytica DSM 15894	1	0	0	0
**63186**	Zobellia galactanivorans	0	0	1	1
