# Supplementary material for: Intergenomic Comparisons Highlight Modularity of the Denitrification Pathway and Underpin the Importance of Community Structure for N2O Emissions
Source: PLoS One. 2014 Dec 1;9(12):e114118. doi: 10.1371/journal.pone.0114118 (PMC4250227; doi:10.1371/journal.pone.0114118)
Supplement: Table S2 — Frequency table of gene co-occurrence patterns and taxonomic affiliation at class level. The nirK (K), nirS (S) and nosZ (Z) co-occurrence types and percentage of organisms within each class that also harbor a nor gene are indicated. (PDF) [file pone.0114118.s007.pdf]

Table S2. Frequency table of gene co-occurrence patterns and taxonomic affiliation at class level. The *nirK* (K), *nirS* (S) and *nosZ* (Z) co-occurrence types and percentage of organisms within each class that also harbor a *nor* gene are indicated.

	K	%nor	KS	%nor	KSZ	%nor	KZ	%nor	S	%nor	SZ	%nor	Z	%nor
Acidimicrobiia	1	0	0	-	0	-	0	-	0	-	0	-	0	-
Actinobacteria	101	43	0	-	0	-	0	-	0	-	0	-	0	-
Alphaproteobacteria	54	59	0	-	0	-	45	98	1	100	14	93	13	31
Anaerolineae	0	-	0	-	0	-	0	-	1	0	0	-	0	-
Aquificae	0	-	0	-	0	-	0	-	0	-	2	100	0	-
Archaeoglobi	0	-	0	-	0	-	0	-	0	-	0	-	1	100
Bacilli	11	36	0	-	0	-	4	100	0	-	1	100	1	0
Bacteroidia	0	-	0	-	0	-	1	100	0	-	0	-	6	0
Betaproteobacteria	35	86	1	100	0	-	34	100	7	86	28	100	3	100
Caldilineae	0	-	0	-	0	-	0	-	0	-	0	-	1	-
Chloroflexi	4	0	0	-	0	-	0	-	1	0	0	-	0	-
Clostridia	5	0	0	-	0	-	0	-	0	-	0	-	7	86
Cytophagia	1	100	0	-	0	-	4	75	0	-	0	-	2	0
Deferribacteres	0	-	0	-	0	-	0	-	0	-	0	-	1	0
Deinococci	0	-	2	100	0	-	0	-	3	100	0	-	0	-
Deltaproteobacteria	2	100	0	-	0	-	0	-	0	-	0	-	5	100
Epsilonproteobacteria	0	-	0	-	0	-	0	-	0	-	6	100	10	60
Eurotiomycetes	22	59	0	-	0	-	0	-	0	-	0	-	0	-
Flavobacteria	7	100	0	-	0	-	16	69	0	-	0	-	10	20
Gammaproteobacteria	44	57	1	100	5	100	17	94	4	100	26	100	16	37
Gemmatimonadetes	0	-	0	-	0	-	1	0	0	-	0	-	0	-
Halobacteria	5	60	0	-	0	-	4	100	0	-	0	-	4	100
Ignavibacteria	0	-	0	-	0	-	0	-	0	-	0	-	2	0
Marine Group I	8	0	0	-	0	-	0	-	0	-	0	-	0	-
Nitrospira	1	0	0	-	0	-	0	-	1	100	0	-	0	-

Opitutae	0	-	0	-	0	-	1	0	0	-	0	-	2	0
Planctomycetacia	1	0	0	-	0	-	0	-	0	-	0	-	0	-
Soil Crenarchaeotic Group	1	0	0	-	0	-	0	-	0	-	0	-	0	-
Sordariomycetes	5	100	0	-	0	-	0	-	0	-	0	-	0	-
Spartobacteria	1	0	0	-	0	-	0	-	0	-	0	-	0	-
Sphingobacteria	0	-	0	-	1	0	1	0	0	-	0	-	2	0
Sphingobacteriia	0	-	0	-	0	-	2	50	0	-	0	-	4	50
Spirochaetes	2	50	0	-	0	-	3	33	0	-	1	100	3	67
Thermomicrobia	1	0	0	-	0	-	1	0	0	-	0	-	1	0
Thermoprotei	0	-	0	-	0	-	0	-	2	100	2	100	0	-
unclassified	2	100	0	-	0	-	0	-	0	-	0	-	0	-

The residuals are according to Pearson's Chi-squared test (P<0.001). Combinations with Pearson residuals >2 are highlighted in blue, whereas combinations with Pearson residuals <-2 are highlighted in red.
