# Supplementary material for: Intergenomic Comparisons Highlight Modularity of the Denitrification Pathway and Underpin the Importance of Community Structure for N2O Emissions
Source: PLoS One. 2014 Dec 1;9(12):e114118. doi: 10.1371/journal.pone.0114118 (PMC4250227; doi:10.1371/journal.pone.0114118)
Supplement: Table S3 — Frequency table of gene co-occurrence patterns and taxonomic affiliation at order level. The nirK (K), nirS (S) and nosZ (Z) co-occurrence types and percentage of organisms within each order that also harbor a nor gene are indicated. (PDF) [file pone.0114118.s008.pdf]

Table S3. Frequency table of gene co-occurrence patterns and taxonomic affiliation at order level. The *nirK* (K), *nirS* (S) and *nosZ* (Z) co-occurrence types and percentage of organisms within each order that also harbor a *nor* gene are indicated.

	**K**	**%nor**	**KS**	**%nor**	**KSZ**	**%nor**	**KZ**	**%nor**	**S**	**%nor**	**SZ**	**%nor**	**Z**	**%nor**
Acidimicrobiales	1	0	0	-	0	-	0	-	0	-	0	-	0	-
Actinomycetales	28	50	0	-	0	-	0	-	0	-	0	-	0	-
Aeromonadales	1	100	0	-	0	-	0	-	0	-	0	-	1	0
Alteromonadales	5	100	0	-	0	-	4	100	0	-	3	67	5	0
Anaerolineales	0	-	0	-	0	-	0	-	1	0	0	-	0	-
Aquificales	0	-	0	-	0	-	0	-	0	-	2	100	0	-
Archaeoglobales	0	-	0	-	0	-	0	-	0	-	0	-	1	100
Bacillales	11	37	0	-	0	-	4	100	0	-	0	-	1	0
Bacteroidales	0	-	0	-	0	-	1	100	0	-	0	-	6	0
Bdellovibrionales	2	100	0	-	0	-	0	-	0	-	0	-	1	100
Bifidobacteriales	6	0	0	-	0	-	0	-	0	-	0	-	0	-
Brocadiales	1	0	0	-	0	-	0	-	0	-	0	-	0	-
Burkholderiales	15	73	1	100	0	-	15	100	6	83	13	100	2	100
Caldilineales	0	-	0	-	0	-	0	-	0	-	0	-	1	0
Campylobacterales	0	-	0	-	0	-	0	-	0	-	4	100	10	60
Cardiobacteriales	0	-	0	-	0	-	2	100	0	-	0	-	0	-
Caulobacterales	3	67	0	-	0	-	0	-	0	-	0	-	0	-
Chloroflexales	3	0	0	-	0	-	0	-	1	0	0	-	0	-
Chromatiales	5	60	0	-	0	-	0	-	0	-	0	-	3	100
Chthoniobacterales	1	0	0	-	0	-	0	-	0	-	0	-	0	-
Clostridiales	5	0	0	-	0	-	0	-	0	-	0	-	7	86
Corynebacteriales	22	41	0	-	0	-	0	-	0	-	0	-	0	-
Cytophagales	1	100	0	-	0	-	4	75	0	-	0	-	2	0
Deferribacterales	0	-	0	-	0	-	0	-	0	-	0	-	1	0
Eurotiales	6	67	0	-	0	-	0	-	0	-	0	-	0	-

Flavobacteriales	7	100	0	-	0	-	16	69	0	-	0	-	10	20
Frankiales	2	0	0	-	0	-	0	-	0	-	0	-	0	-
Gemmatimonadales	0	-	0	-	0	-	1	0	0	-	0	-	0	-
Halobacteriales	5	60	0	-	0	-	4	100	0	-	0	-	4	50
Herpetosiphonales	1	0	0	-	0	-	0	-	0	-	0	-	0	-
Hydrogenophilales	0	-	0	-	0	-	0	-	0	-	1	100	0	-
Ignavibacteriales	0	-	0	-	0	-	0	-	0	-	0	-	2	0
Lactobacillales	0	-	0	-	0	-	0	-	0	-	1	100	0	-
Methylococcales	0	-	0	-	1	100	0	-	0	-	0	-	0	-
Methylophilales	1	100	0	-	0	-	0	-	0	-	0	-	0	-
Micrococcales	16	44	0	-	0	-	0	-	0	-	0	-	0	-
Micromonosporales	4	0	0	-	0	-	0	-	0	-	0	-	0	-
Myxococcales	0	-	0	-	0	-	0	-	0	-	0	-	4	100
Nautiliales	0	-	0	-	0	-	0	-	0	-	2	100	0	-
Neisseriales	14	100	0	-	0	-	19	100	0	-	2	100	0	-
Nitrosomonadales	5	80	0	-	0	-	0	-	1	100	0	-	0	-
Nitrospirales	1	0	0	-	0	-	0	-	1	100	0	-	0	-
Oceanospirillales	1	100	1	100	0	-	1	100	0	-	1	100	1	0
Onygenales	15	53	0	-	0	-	0	-	0	-	0	-	0	-
Opitutales	0	-	0	-	0	-	1	0	0	-	0	-	2	0
Pasteurellales	17	41	0	-	0	-	0	-	0	-	0	-	0	-
Propionibacteriales	16	75	0	-	0	-	0	-	0	-	0	-	0	-
Pseudomonadales	11	54	0	-	4	100	2	100	4	100	20	100	1	100
Pseudonocardiales	2	0	0	-	0	-	0	-	0	-	0	-	0	-
Rhizobiales	44	57	0	-	0	-	33	97	0	-	1	0	6	17
Rhodobacterales	5	60	0	-	0	-	10	100	1	100	11	100	5	40
Rhodocyclales	0	-	0	-	0	-	0	-	0	-	12	100	1	100
Rhodospirillales	1	100	0	-	0	-	2	100	0	-	2	100	2	50
Salinisphaerales	0	-	0	-	0	-	1	100	0	-	0	-	0	-
Sphaerobacterales	1	0	0	-	0	-	0	-	0	-	0	-	0	-

Sphaerobacteridae	0	-	0	-	0	-	1	0	0	-	0	-	0	-
Sphingobacteriales	0	-	0	-	1	0	3	33	0	-	0	-	6	33
Sphingomonadales	1	100	0	-	0	-	0	-	0	-	0	-	0	-
Spirochaetales	2	50	0	-	0	-	3	33	0	-	1	100	3	67
Streptomycetales	2	50	0	-	0	-	0	-	0	-	0	-	0	-
Streptosporangiales	3	0	0	-	0	-	0	-	0	-	0	-	0	-
Thermales	0	-	2	100	0	-	0	-	3	100	0	-	0	-
Thermomicrobiales	0	-	0	-	0	-	0	-	0	-	0	-	1	0
Thermoproteales	0	-	0	-	0	-	0	-	2	100	2	100	0	-
Thiotrichales	1	0	0	-	0	-	0	-	0	-	0	-	1	100
unclassified	17	47	0	-	0	-	0	-	0	-	1	100	0	-
Vibrionales	0	-	0	-	0	-	0	-	0	-	1	100	4	25
Xanthomonadales	3	67	0	-	0	-	7	86	0	-	0	-	0	-

The residuals are according to Pearson’s Chi-squared test (P<0.001). Combinations with Pearson residuals >2 are highlighted in blue, whereas combinations with Pearson residuals <-2 are highlighted in red.
